# Supervised 16-Week Multicomponent Exercise Training Programme for 18–55-Year-Old People Living with and Beyond Cancer—CONSORT 2025-Based Study Protocol of the Pilot Onco-Move Randomized Controlled Trial

**DOI:** 10.3390/nu18010100

**Published:** 2025-12-27

**Authors:** Marzena Wieczorek-Przybyło, Milena Lachowicz, Maja Tomczyk, Agnieszka Kowalska, Mateusz Sprengel, Jakub Szczubełek, Michał Niedźwiecki, Wojciech Barton, Bartłomiej Zyborowicz, David Jiménez-Pavón

**Affiliations:** 1 Department of Psychology, Gdansk University of Physical Education and Sport, 80-336 Gdansk, Poland; 2 Department and Clinic of Oncology and Radiotherapy, Medical University of Gdansk, 80-210 Gdansk, Poland; milena.lachowicz@gumed.edu.pl; 3 Department of Biochemistry, Gdansk University of Physical Education and Sport, 80-336 Gdansk, Poland; maja.tomczyk@awf.gda.pl (M.T.); agnieszka.kowalska@awf.gda.pl (A.K.); 4 Department of Physiology, Gdansk University of Physical Education and Sport, 80-336 Gdansk, Poland; mateusz.sprengel@awf.gda.pl (M.S.); michalniedwied@gmail.com (M.N.); wojciech.barton@awf.gda.pl (W.B.); 5 Independent Researcher, 80-000 Gdansk, Poland; jakubszczubelek@wp.pl (J.S.); zyborowiczb@gmail.com (B.Z.); 6 MOVE-IT Research Group, Department of Physical Education, Faculty of Education Sciences and Biomedical Research Innovation Institute of Cádiz, University of Cádiz, 11519 Cadiz, Spain; david.jimenez@uca.es

**Keywords:** cancer, cancer exercise, exercise, cancer survivors, physical activity interventions, exercise programmes, quality of life

## Abstract

Despite evidence that exercise improves treatment outcomes, most people living with and beyond cancer (PLWBC) are insufficiently physically active. To address this challenge the Onco-Move Improvement of psychophysical fitness in adult cancer survivors pilot randomized controlled trial aims to investigate the 7-month effects of a 16-week supervised, multicomponent exercise programme on PLWBC adults aged 18–55. The study will include 40 participants currently undergoing cancer treatment or up to 5 years post-treatment with different types of cancer, who will be randomly assigned to the experimental or the control group. The exercise programme will take place three times a week for 16 weeks. We hypothesize that the Onco-Move exercise programme will improve participants’ quality of life, sexual function, and stress-response strategies, as well as their nutrition, physical functions, and body composition. We also expect to observe physiological changes related to the intervention influencing inflammation, metabolism, muscle adaptation, and cellular aging. The Onco-Move exercise programme, once validated, has the potential to be implemented in oncological non-governmental organizations (NGOs) and hospitals in Poland and Spain, among others, as a feasible, effective, and safe non-pharmacological aid for cancer patients and survivors.

## 1. Introduction

Cancer accounted for nearly 20 million new cases and 10 million deaths in 2022 [1]. Estimates suggest that approximately one in five men or women will develop cancer in their lifetime, whereas around one in nine men and one in twelve women will die from it [1]. The number of diagnoses is also increasing among younger adults [2]. One-fourth of new incidents in 2022 occurred among people aged 15–54, accounting for nearly 1.7 million deaths in this population [3]. Importantly, early-onset cancer (defined as cancer diagnosed before the age of 50) has been rising globally in recent decades. At the same time, the majority of published exercise oncology research has focused on older adults, leaving younger and middle-aged people living with and beyond cancer relatively underrepresented in clinical trials. Moreover, projections indicate that the global incidence and mortality of early-onset cancer are expected to increase by 31% and 21%, respectively, by 2030 [4].

Advanced medical treatments have significantly reduced mortality rates among cancer patients [5]. However, the majority of individuals living with and beyond cancer will experience compromised physical and cognitive function due to cancer treatments and side-effects [6]. Treatments such as chemotherapy, radiotherapy, immunotherapy, or surgery can have harmful side-effects that negatively impact quality of life [7]. Physical activity can be an answer to avoid or minimize some of these side-effects.

In the past, clinicians advised cancer patients to rest and to avoid physical activity, but early exercise research in the 1990s and 2000s challenged this advice [5]. Between 2010 and 2022, the American Cancer Society (ACS) and the American College of Sports Medicine (ACSM) published recommendations for physical exercise and nutrition for cancer survivors [5,8,9]. Similarly, in 2021, the World Health Organization (WHO) released guidelines asserting that physical activity can benefit individuals post-cancer treatment by reducing mortality from cancer and lowering the risk of recurrence or the emergence of a second primary cancer [10].

Despite evidence that exercise improves treatment outcomes, PLWBC do not follow these recommendations. A total of 36.7% of cancer survivors aged 18 years and older do not perform any physical activity [11], and up to 93% report insufficient physical activity in their leisure time [12]. Some contributing factors include a lack of time, motivation, and self-discipline and also a lack of personalized exercise programs [12].

To address these challenges, the Onco-Move pilot randomized controlled trial aims to investigate the 7-month effects of a 16-week supervised, multicomponent exercise programme on adults aged 18–55 living with or beyond cancer. The results of this study may help to assess the effectiveness of exercise as a therapeutic component of health-related quality of life (HRQoL), functional fitness, body composition, nutrition, and other factors described later in this publication.

Although no dietary intervention will be implemented, nutrition is considered a core lifestyle dimension within the Onco-Move study. Adherence to the Mediterranean diet will be systematically assessed using the validated MEDAS questionnaire, not only as a control variable but also as a relevant lifestyle factor that may interact with exercise-related adaptations in PLWBC. This feasibility-oriented, observational approach allows for the exploration of diet–exercise interactions in real-world conditions, providing preliminary insight to inform future combined exercise–nutrition interventions.

## 2. Materials and Methods

### 2.1. Study Design and Protocol Registration

The Onco-Move pilot project is a parallel two-arm randomized controlled trial (RCT). The research aims to gain knowledge about a 16-week multicomponent in-person exercise programme and to investigate the extent to which it supports PLWBC, aged 18–55, both psychologically and functionally during the treatment process and after its completion, as well as whether it has an impact on long-term effects. The intervention is reported according to the Consensus on Exercise Reporting Template (CERT).

The study was registered at www.clinicaltrials.gov (NCT07087652) on 28 July 2025, before the enrolment of participants. The design of the project and the study protocol follow the CONSORT 2025 reporting guidelines [13].

### 2.2. Study Setting

Gdansk University of Physical Education and Sport (GUPES), Poland, will be responsible for the coordination and delivery of the Onco-Move project, which is co-designed in cooperation with the MOVE-IT Research group from the University of Cádiz, Spain. The research, intervention and monitoring will take place at GUPES.

### 2.3. Study Design

**Figure 1:**
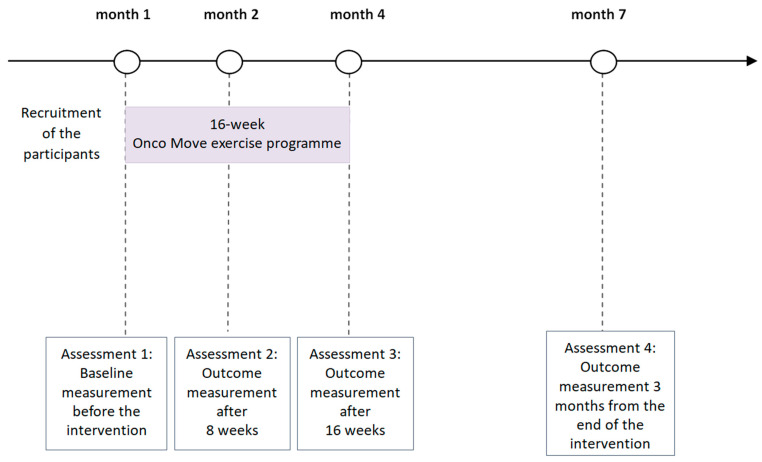
Data collection diagram.

The project timeline is presented in Figure 1.

### 2.4. Recruitment

Recruitment will be supported by oncological foundations and associations from the Pomeranian Voivodeship and the University Clinical Center in Gdansk. A social media campaign will be implemented, and a project website will be published: https://onkotrening.pl/onco-move/ (accessed on 13 October 2025). Moreover, the project brochure will be distributed in hospitals and to oncological NGOs.

### 2.5. Eligibility Criteria

The participants’ inclusion and exclusion criteria are listed in Table 1.

In the case of pregnant women who wish to participate in the project, they will not be enrolled in the intervention but will be allowed to attend a free educational lecture on the WHO and ACSM guidelines for recommended physical activity for cancer patients.

### 2.6. Sample Size

The study was pragmatically powered with n = 20 participants per group (intervention and control) for this initial feasibility and estimation phase. The calculation assumes a two-sided α = 0.05 and a nominal power of 80%, using an ANCOVA model adjusted for baseline values and repeated measures to increase statistical efficiency. Previous exercise-based trials in PLWBC [14,15,16] have reported between-group differences of approximately 8–10 points in the EORTC QLQ-C30 Global Health Status/QoL domain, with standard deviations around 20–30 points. Based on these data, a fully powered confirmatory trial would require substantially larger samples; therefore, the present study adopts a realistic initial sample size aimed at (i) quantifying the signal of efficacy and its heterogeneity across subgroups, (ii) evaluating feasibility, adherence, and safety, and (iii) providing empirical estimates of variance, correlation, and attrition rates to inform a subsequent definitive trial with higher precision. Should recruitment capacity allow, the sample may be progressively expanded to narrow confidence intervals around clinically meaningful differences of 8–10 points.

Furthermore, feasibility expectations regarding adherence and dropout are informed by a previous pilot study conducted in 2023 at GUPES. Based on this experience, we anticipate an adherence rate of approximately 60–70% in the current RCT, with an expected dropout rate of around 25%. Dropout is primarily anticipated among participants undergoing active cancer treatment, who may experience treatment-related side-effects or changes in medical status that prevent continued participation.

### 2.7. Randomization

Participants will be randomly allocated to either the experimental group (EG) or the control group (CG), with n = 20 participants per group. The random sequence will be computer-generated in blocks of four to ensure balanced allocation between groups throughout recruitment. The sequence will be generated by the project coordinator at the University of Cádiz (MOVE-IT Research Group, Spain), who is not involved in participant recruitment, assessment, or intervention delivery, thereby ensuring allocation concealment. Group assignments will be communicated to the local principal investigator, who will subsequently inform each participant of their allocation. This procedure minimizes selection bias and preserves blinding of outcome assessors and data analysts.

### 2.8. Blinding

Due to the nature of the intervention, participants and exercise trainers cannot be blinded. The study will therefore adopt a single-blind design, in which outcome assessors and data analysts remain blinded to group allocation. The randomization sequence will be stored by an independent investigator not involved in assessments or analyses. Participants will be instructed not to disclose their allocation during assessments. This approach ensures methodological transparency while minimizing assessment bias.

### 2.9. Assessment Protocol

Each patient who participates in the project will undergo four assessments over the course of the project: (1) a baseline assessment—before participating in the Onco-Move training programme; (2) 8 weeks after the programme begins; (3) 16 weeks after the programme begins; (4) 3 months after the completion of the Onco-Move intervention.

### 2.10. Outcomes

Given the pilot and feasibility-oriented design of the study, health-related quality of life (HRQoL) is defined as the sole primary outcome. All other outcomes, including functional, physiological, biochemical, genetic, and telomere-related measures, are considered secondary or exploratory and will be interpreted with an emphasis on estimation, feasibility, and hypothesis generation rather than confirmatory inference.

**Figure 2:**
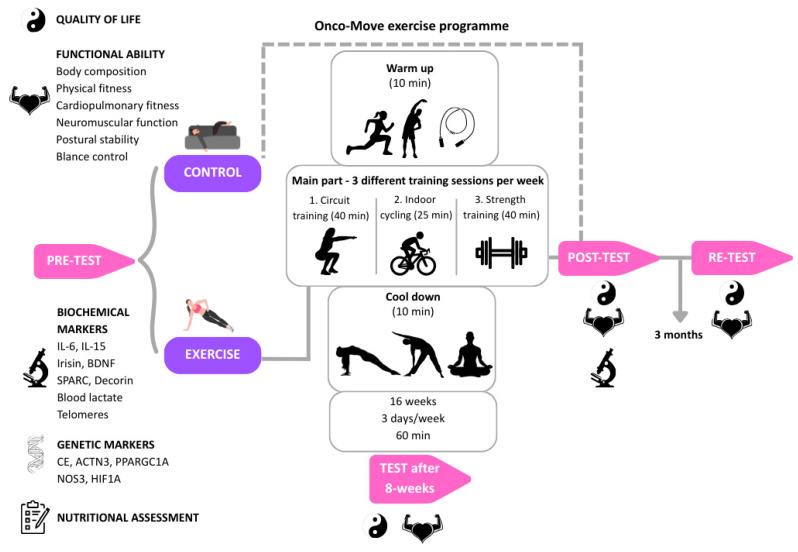
Overview of the Onco-Move study design and exercise intervention. The figure illustrates the timeline of assessments and the structure of the Onco-Move exercise programme. Participants will undergo baseline (pre-test) assessments, followed by a 16-week supervised exercise intervention (3 sessions per week), with an intermediate assessment after 8 weeks, a post-intervention assessment (after 16 weeks), and a 3-month follow-up (re-test). Icons represent the main outcome domains assessed at each time point, including quality of life, functional ability, biochemical and genetic markers, and nutritional assessment. Arrows indicate the chronological flow of the intervention and assessments. The central panel depicts the structure of each training session, including warm-up, main training components, and cool-down.

A graphical design of the outcomes measured is presented in Figure 2.

**a. Primary outcome**

1. *HRQOL* will be assessed pre-, during, and post-intervention and at follow-up using a validated questionnaire: EORTC QLQ-C30 [17]. This instrument measures general quality of life and includes multiple subscales (e.g., physical, emotional, cognitive functioning). Scores on each scale range from 0 to 100. For functional scales and global health status, higher scores indicate better functioning or QoL. For symptom scales, higher scores indicate more severe symptoms. The validated Polish version of the EORTC QLQ-C30 will be used. Data will be collected via digital or paper formats and analyzed.

**b. Secondary outcomes**

1. *Other quality-of-life (QoL) variables*—Other QoL-related variables will be assessed pre-, during, and post-intervention and at follow-up using validated questionnaires. Scores will be calculated per standard procedures. Data will be collected via digital or paper formats and analyzed. Tools include the following: (A) *Female Sexual Function Index (FSFI)* [18]—This is a 19-item validated questionnaire measuring female sexual function across six domains: desire, arousal, lubrication, orgasm, satisfaction, and pain. Scores range from 2 to 36, with higher scores indicating better sexual function. (B) *International Index of Erectile Function (IIEF)* [19]—This is a 15-item questionnaire assessing male sexual function, including erectile function, orgasmic function, sexual desire, intercourse satisfaction, and overall satisfaction. Scores range from 5 to 75, with higher scores indicating better sexual function. (C) *UWIST Mood Adjective Checklist (UMACL)* [20]—The questionnaire measures three mood dimensions: tense arousal, energetic arousal, and hedonic tone. Scores vary by subscale; higher scores indicate greater presence of the respective mood state. (D) *The Feeling of Stress Questionnaire (KPS)* [21]—The tool assesses perceived psychosocial stress across domains such as work and emotional strain. Scores vary by domain; higher scores indicate greater perceived stress. (E) *Patient Health Questionnaire For Anxiety And Depression (PHQ-9)* [22]—This is a 9-item screening tool with two items each for anxiety and depression. Scores range from 0 to 12, with higher scores indicating more severe symptoms. (F) *NEO Five-Factor Inventory (NEO-FFI)* [23]—This assesses five personality traits: neuroticism, extraversion, openness, agreeableness, and conscientiousness. Each trait is scored separately; higher scores indicate stronger presence of the trait. (G) *State–Trait Anxiety Inventory (STAI)* [24]—This includes two 20-item subscales measuring state anxiety (temporary) and trait anxiety (long-term). Scores range from 20 to 80 per subscale, with higher scores indicating greater anxiety. All questionnaires will be administered in their validated Polish-language versions.
2. *Adherence to the Mediterranean diet*—Adherence to a Mediterranean dietary pattern will be assessed using the 14-item Mediterranean Diet Adherence Screener (MEDAS), originally developed and validated in the PREDIMED trial [25]. This questionnaire evaluates the frequency of consumption of key food components characteristic of the Mediterranean diet, such as olive oil, fruits, vegetables, legumes, nuts, fish, wine, and limits on red meat and processed foods.
3. *Body composition*—This will be measured using a bioimpedance analyzer (InBody 720, InBody Co., Ltd., Seoul, Republic of Korea), including weight (kg), body fat (%), fat mass (kg), fat-free mass (kg), bone mass (kg) intra-extracellular water (L), and waist-to-hip ratio (WHR), among others. Body composition will be assessed using the InBody 720 bioimpedance analyzer [26]. The participants will be asked to fast for at least 2 h and to urinate before the assessment. Measurements will be taken at baseline, during and post-intervention, and at follow-up to evaluate physiological changes related to the intervention.
4. *Self-reported physical fitness (IFIS)*—The International Fitness Scale (IFIS) [27] is a subjective self-assessment tool for evaluating perceived physical fitness.
5. *Physical activity objectives*—Participants’ motivation for participating in the project will be measured by the Inventory of Physical Activity Objectives (IPAO) [28]. IPAO contains four scales of goal-oriented behaviours associated with physical activity (PA): (1) motivational value, (2) time management, (3) persistence in action, and (4) motivational conflict.
6. *Physical function*—This will be evaluated using two adapted fitness test batteries: the *Senior Fitness Test (SFT)* [29] and the *Short Physical Performance Battery (SPPB)* [30], tailored to the needs and limitations of the participants. From the SFT, we will measure key aspects of physical fitness relevant to daily activities: lower limb strength (chair rise test), upper limb strength (arm curl test), and aerobic capacity (6 min walk). The SPPB will include lower limb strength (chair stand test). Both test sets help monitor changes in physical fitness, assess fall risk, and guide rehabilitation planning. Additionally, we will test the walking speed (fast) by a 10-Metre Fast Walking Test (10MWT). Measurements will be taken at baseline, during and post-intervention, and at follow-up.
7. *Postural stability and balance control*—Balance and postural stability will be assessed using the BIODEX Stabilometric Platform (Biodex Medical Systems, Inc., Shirley, NY, USA) [31]. This device objectively measures static and dynamic balance, overall stability, and controlled mobility. Measurements will be taken at baseline, during and post-intervention, and at follow-up to monitor changes in postural control and balance performance. Participants will stand barefoot on a platform so that their centre of gravity is positioned in the centre of the platform. The researcher will record the participant’s foot position and will conduct four types of tests: on a stable surface and on a moving surface, with open and closed eyes. After all the measurements are completed, a report of the conducted test will be generated.
8. *Neuromuscular function* (isokinetic dynamometry)—Neuromuscular performance will be evaluated using the Biodex System 4 Pro (Biodex Medical Systems, Inc., Shirley, NY, USA) [32], an advanced dynamometric system for assessing and training muscle function under various contraction modes. The device allows for testing in isometric, isotonic (concentric and eccentric), isokinetic (concentric and eccentric), reactive eccentric, and passive motion conditions. It provides objective data on muscle strength, endurance, and control, with full data archiving and export capabilities for statistical analysis. Measurements will be conducted at baseline, during and post-intervention, and at follow-up to detect changes in neuromuscular function over time. Participants will sit on a specialized chair. The limb being tested will be secured in a movable panel connected to a dynamometer measuring force. Participants will be instructed on the direction of movement, after which they will perform several trial repetitions. During the protocol, data will be acquired by the device and displayed on the monitor screen. Isokinetic neuromuscular assessment using the Biodex system will be conducted only in participants without specific contraindications. Exclusion criteria for this assessment include known or suspected bone metastases, presence of central venous catheters (e.g., PICC lines), recent surgery affecting the tested limb, acute treatment-related complications, or any medical contraindication identified by the treating oncologist. Participants meeting these criteria will be excluded from isokinetic testing while remaining eligible for other study assessments.
9. *Cardiopulmonary fitness* (ergospirometry)—Cardiopulmonary capacity will be evaluated using a Vyntus CPX (Vyaire Medical, Inc., Mettawa, IL, USA) [33], which analyzes exhaled gases during exercise to assess respiratory and circulatory function. Key parameters measured include maximum oxygen uptake (VO_2_ max), peak oxygen uptake (VO_2_ peak), ventilatory response to exercise (VE/VCO_2_ slope), and the respiratory exchange ratio (RER = VCO_2_/VO_2_), which is useful for evaluating anaerobic metabolism during exercise. These measurements will be taken on a cycle ergometer at baseline, during and post-intervention, and at follow-up to assess improvements in cardiorespiratory fitness. The participants will be asked to avoid caffeine before the test. During the test, participants exert themselves with increasing intensity, and respiratory and metabolic parameters are monitored and recorded. The protocol for the incremental test on the cycle ergometer will include familiarization with the equipment (minutes 0–3); warm-up at 0.5 W/kg (minutes 3–6); from minute 6 onward, an increase of +0.25 W/kg per minute; and a cool-down period (last 3 min).
10. *Blood lactate levels*—Blood lactate concentration will be measured using the Lactate Scout 4 system (EKF Diagnostics GmbH, Barleben, Germany) [34]. This device is designed for assessing lactate levels in capillary blood, providing insights into changes in physical fitness and endurance. Measurements will be taken at baseline and post-intervention to evaluate improvements in physical performance and metabolic response during exercise. Lactate levels will be measured via an ear prick during the exercise test. A baseline assessment will be performed before the start of the test, and then levels will be checked at the 5th minute 1 min into the cool-down, and 5 min after the end (lactate levels will be tested four times during each test).
11. *Grip strength*—Grip strength will be measured using a SAEHAN electronic dynamometer (Saehan Corporation, Changwon, Republic of Korea) [35]. This device evaluates handgrip strength by measuring grip force in an isometric test. The dynamometer features a five-level adjustable grip (ranging from 3.4 cm to 8.5 cm) to accommodate different hand sizes. The measurements will be taken at baseline, during and post-intervention, and at follow-up to track changes in muscle strength and hand function. Participants will squeeze the dynamometer according to the instructions provided by the researcher.
12. *Biochemical markers*—Biochemical markers will be measured from participants’ blood at baseline and post-intervention. The markers include IL-6 (Interleukin-6), IL-15 (Interleukin-15), Irisin, BDNF (Brain-Derived Neurotrophic Factor), SPARC (Secreted Protein, Acidic, and Rich in Cysteine), and Decorin. These biomarkers will provide insights into the participants’ inflammatory, metabolic, and muscular responses to the intervention, allowing for the assessment of physiological changes related to the intervention.
13. *Genetic analyses* will be conducted using participants’ blood samples collected at baseline. The analysis will include polymorphisms located in the following genes: ACE, ACTN3, PPARGC1A, BDNF, VEGF, IL6, NOS3, HIF1A, TNF-α, and mTOR pathway. These genetic markers will help evaluate individual variability in response to the intervention and provide insights into genetic influences on inflammation, metabolism, muscle adaptation, and cellular aging. Given the limited sample size, the collected samples will be stored and reserved for future pooled analyses.
14. *Telomeres and telomerase*—Blood samples will be collected at baseline and after 16 weeks of the Onco-Move training. All samples from the same participant will be analyzed on the same plate to ensure consistency, which is crucial for scientific validation. Results will be expressed as relative telomere length.
15. *Post-training assessment of Onco-Move exercise programme*—Participants within 24 h from a training session will report their psychophysical well-being online via a Google document form.

We are aware that the assessment battery may be demanding. Therefore, all tests were carefully selected based on their clinical relevance and feasibility, and the total duration of each assessment session was evaluated in advance. The full testing protocol will be pilot-tested prior to study initiation, and the research team will be extensively trained to conduct all assessments as efficiently as possible in order to minimize participant fatigue and burden. Assessments will be scheduled with appropriate rest periods, and procedures may be adapted or postponed if participants experience excessive fatigue or treatment-related symptoms.

A summary of the time-point measurements is presented in Table 2.

### 2.11. Control Variables and Other Parameters to Be Recorded

Different variables will be recorded that may influence the study results, such as (1) sex, age, educational level, marital status, occupational status, the place of living (a city or the countryside); (2) type of cancer; (3) type of cancer treatment (surgery, chemotherapy, immunotherapy, targeted therapy, radiotherapy, or other); and (4) current treatment status (before, during or after the treatment).

### 2.12. Interventions

**Figure 3:**
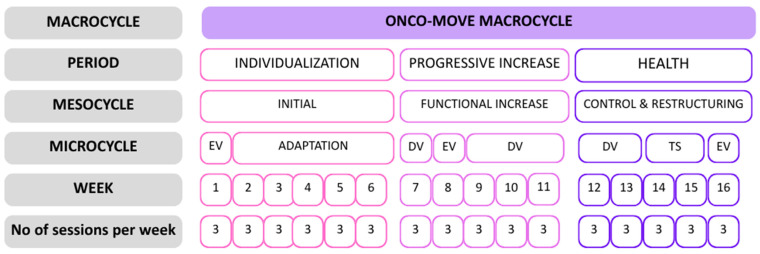
The figure illustrates the 16-week training macrocycle structured into periods, mesocycles, and microcycles, following an individualized and progressive approach. EV indicates evaluation phases, DV represents development phases, and TS denotes a transitional stimulus applied between training phases. The programme includes three supervised sessions per week throughout the intervention.

The Onco-Move exercise intervention is planned according to the CERT. The programme is based on the Individualized and Progressive Health Model [36] (presented in Figure 3.: Onco-Move Individualized and Progressive Health Model) and will include different types of exercises covering all the fitness components (cardio, strengthening, balance, shaping exercises, and stretching). The Individualized and Progressive Health Model (IPS) is a conceptual framework described in a technical monograph [36] and grounded in established exercise prescription principles widely supported by peer-reviewed exercise oncology guidelines.

Participants will perform exercises without any equipment, whereas specific exercises will be performed with such equipment as sports rubber bands, sensory-motor discs, Pilates balls, dumbbells, and cycle ergometers.

All training sessions will be conducted in fitness studios and the gym at Gdansk University of Physical Education and Sport by four certified professional fitness instructors, who will be additionally trained by a certified cancer exercise specialist, and the entire exercise program will be supervised by an expert exercise physiologist with experience in exercise interventions. The in-person interventions will be provided to a group of 20 participants.

The instructors who will supervise exercise performance will correct exercise techniques, ensure the participants maintain the correct movement pattern, provide guidance, provide feedback, and modify exercises as required by a specific type of cancer and treatment.

Adherence will be measured by the percentage of the Onco-Move training sessions completed, defined as the number of the training sessions attended divided by the total number of the training sessions delivered. The reason for absence from training (caused by the treatment regimen, side-effects of treatment, infections, and other reasons) will be reported by patients completing the post-training assessment form (an online tool created by the research team on the Google Forms platform). This approach will allow for feasibility to be evaluated under real-world conditions and provides critical information on barriers that cannot be fully eliminated within the scope of the current study.

Participants who take part in the training will report on the post-training assessment form the extent to which the training affects their general wellbeing. Online post-training assessment forms will be intentionally brief, mobile-friendly, and designed to be completed within a short time frame to reduce participant burden.

Several motivational and social support strategies will be embedded in the Onco-Move intervention. A dedicated WhatsApp group will serve as the primary communication platform to provide timely updates regarding training sessions, assessments, and organizational matters, as well as motivational messages and reminders. This channel will also facilitate peer interaction and social support to enhance adherence in supervised exercise programmes for PLWBC. To strengthen group cohesion and accountability, team-building elements will be incorporated, including group photos, short motivational videos documenting progress, and collective celebration of milestones and seasonal events (e.g., holidays). These activities will be intended to foster a sense of belonging and shared commitment, which may help mitigate dropout risk in a demanding programme.

With respect to logistical burden, training sessions will be scheduled at fixed and predictable time slots to support routine formation while maintaining limited flexibility, when possible, to accommodate treatment schedules and fatigue. All training sessions and assessments will be conducted at a single, familiar location, thereby minimizing logistical complexity and travel-related burden.

Supplementary Materials are provided in Table S2, with a comprehensive description of the Onco-Move exercise programme comprehensive table with photographs for each exercise, videos on the exercises available on https://onkotrening.pl/onco-move-exercise-programme/ (accessed on 13 October 2025), and the list of equipment in Table S3.

Briefly, the training sessions will be conducted in a large enough fitness studio, where each participant will have enough space to exercise. Those participants who report balance issues will be supported by chairs.

The programme will take place three times a week for 16 weeks. Each session will last 45–60 min (fitness and strength training: 10’ warm-up, 40’ training, 10’ stretching, and interval indoor cycling: 10’ warm-up, 25’ training, 10’ stretching).

The exercise programme will be generic for all PLWBC participants and will follow the Exercise Guidelines for Cancer Survivors from the American College of Sports Medicine [5]. However, fitness instructors will be able to modify the exercise prescription based on the patient’s response to the exercise, type of cancer, and treatment and side-effects related to them. Exercise intensity prescription and progression will be primarily based on peak heart rate obtained during cardiopulmonary exercise testing. Given the potential limitations of threshold determination and VO_2_ max assessment in deconditioned cancer populations, training intensity will be individualized using heart rate reserve (HRR), calculated according to the Karvonen method. Target training zones will be expressed as percentages of HRR and progressively adjusted throughout the intervention period according to predefined progression ranges, participant tolerance, perceived exertion, and clinical status.

All adverse events related to the project, intervention, and study measurements will be recorded.

The four fitness instructors will attend a five-hour training session covering knowledge about cancer types, treatment, possible side-effects, and delivery of the Onco-Move exercise programme. Monthly meetings will be held to discuss issues experienced during the training sessions, and solutions will be suggested.

Before the project starts, a risk analysis will be carried out in accordance with the methodology of the Project Management Institute. An impact-probability matrix will be created to identify the project’s most serious risks and conduct a qualitative risk analysis. The most serious risks identified are participant dropout from the Onco-Move programme for health or other reasons and participant safety—procedures to follow should a patient sustain an injury while participating in the proposed training programme. Appropriate risk management strategies will be applied to minimize these risks.

Participant safety will be ensured at multiple levels. First, all participants will be required to obtain written approval from their oncologist prior to enrolment. Baseline functional assessments will be conducted before participation, and exercise prescriptions will be progressively adapted according to participants’ clinical status, treatment phase, and observed tolerance.

During all supervised training sessions, participants will be continuously monitored by certified fitness instructors. Clear criteria for temporary exercise cessation will be applied, including, but not limited to, chest pain, dizziness, excessive dyspnoea, abnormal fatigue, nausea, musculoskeletal pain, or any symptom perceived as alarming by the participant or instructor. In such cases, exercise will be immediately stopped, vital signs will be assessed, and the participant will be referred for medical evaluation when indicated.

Criteria for discontinuation from the intervention will include medical contraindications identified by the treating physician, significant worsening of clinical status, serious adverse events related to exercise, or participant withdrawal of consent. All adverse events will be documented and classified according to severity and relatedness to the intervention.

Importantly, as this study is designed as a feasibility-oriented RCT, safety outcomes (including adverse events, reasons for missed sessions, temporary exercise interruption, and withdrawal) will be treated as key feasibility indicators. These data will inform the refinement of safety protocols and stopping rules in subsequent larger-scale trials.

### 2.13. Data Collection, Management, and Analysis

On the day of the first measurements at the Physical Exercise Laboratory at GUPES, project participants will be given individual codes according to the scheme n/01/OM, where n means the participant’s consecutive number, 01—first edition, and OM—Onco-Move program. Data typical for each of the research tasks will be collected in Excel spreadsheets. These data will be successively entered into a collective electronic database. Standardized coding of participants will enable easy linking of databases, including, e.g., performance test results and their multiple use.

The data will be stored in the form of Excel and Statistic electronic spreadsheets, Word documents of measurements, and recordings of exercise sessions (jpg, tiff, mp4 format). The data will be also stored in paper form. Data in paper form will be collected in an amount proportional to the number of project participants and stored in properly described binders. Documents will be classified and described according to the procedures adopted by GUPES.

Moreover, the consent form for participation will be distributed to all participants and signed.

Personal and sensitive data will be secured in accordance with the regulations in force at GUPES, with particular attention to data safety. Personal data will be processed and protected in accordance with the Act of 10 May 2018 on the protection of personal data and the guidelines applicable at GUPES.

### 2.14. Statistical Analysis

Descriptive statistics will summarize baseline characteristics. Primary analyses will follow the intention-to-treat (ITT) principle. To evaluate changes across time points (baseline, 8 weeks, 16 weeks, follow-up), a mixed-effects model for repeated measures (MMRM) will be applied with random intercepts for participants and fixed effects for group, time, and group × time interaction, adjusting for relevant covariates (baseline value, age, sex, treatment status). A covariance structure such as compound symmetry or AR(1) will be selected based on model fit.

For outcomes with only two time points or in case of convergence limitations due to sample size, ANCOVA models adjusted for baseline values will be used as a complementary approach. Effect sizes (Hedges’ g) and 95% confidence intervals will be reported to prioritize estimation over strict hypothesis testing, in line with the feasibility nature of the study. Missing data will be handled using maximum likelihood estimation within the mixed-model framework, and complete-case or multiple-imputation sensitivity analyses will be performed when appropriate. Exploratory moderator analyses (e.g., treatment status, cancer type, adherence) will be reported descriptively. Data will be analyzed using IBM SPSS Statistics (version 29) and Stata (version 18). All tests will be two-sided with α = 0.05.

Given the pilot and feasibility-oriented nature of the study, formal correction for multiple comparisons is not planned. Secondary and exploratory outcomes will be interpreted with caution, emphasizing estimation, variability, and hypothesis generation rather than confirmatory statistical inference.

### 2.15. Expected Results

We expect that participation in the Onco-Move programme will lead to improvements in key psychophysical outcomes, including health-related quality of life, emotional well-being, stress-response strategies, and physical functioning. We also anticipate positive adaptations in neuromuscular performance, functional fitness, balance, cardiorespiratory capacity, and body composition. Furthermore, physiological changes are expected in markers of inflammation, metabolism, muscle remodelling, and cellular ageing, reflecting the biological impact of structured exercise.

Regarding nutrition, although no dietary intervention is included, adherence to the Mediterranean diet will be assessed before and after the programme using the MEDAS questionnaire. This will allow us to adjust for any incidental changes in dietary habits that could influence the effects attributed to exercise. At the same time, collecting this information will enable exploratory cross-sectional analyses examining how Mediterranean diet adherence relates to other dimensions measured in the project—such as functional fitness, inflammatory biomarkers, psychological health, and overall quality of life. These analyses may provide additional insight into the broader lifestyle context influencing the health of people living with and beyond cancer.

## 3. Discussion

As cancer treatments continue to improve, an increasing number of people will survive and thrive beyond cancer [5]. Notably, the burden that PLWBC place on the economic, social, and health system is striking [37,38,39]. Structured and tailored cancer exercise programmes, like Onco-Move, have the potential to increase survivors’ quality of life and functional ability, supporting them in continuing working and social life interrupted by cancer treatment.

Our project will primarily investigate the effectiveness of the Onco-Move exercise programme on the quality of life of cancer survivors. More importantly, we will be able to test whether the programme affects multiple health-related outcomes, such as functional ability, strength, and quality of life. Moreover, biochemical and genetic markers will help us evaluate individual variability in response to the intervention and provide insights into biochemical and genetic influences on inflammation, metabolism, muscle adaptation, and cellular aging. Additionally, this project will be able to describe how nutrition pattern is related to all these dimensions in PLWBC.

### Limitations

The Onco-Move project is designed as a pilot study and will include a relatively small sample of forty participants with different types of cancer, who are either currently undergoing treatment or up to five years post-treatment. We acknowledge that both the limited sample size and the clinical heterogeneity of the participants constitute important limitations and may reduce the signal-to-noise ratio, particularly for biological outcomes. However, the primary aim of this pilot study is to assess the feasibility, safety, and acceptability of the Onco-Move training programme rather than to draw definitive conclusions regarding physiological mechanisms. Importantly, this study will provide valuable preliminary insights into feasibility parameters, participant adherence, training preferences, and perceived barriers, which will inform the design of future, adequately powered trials with more homogeneous subgroups or stratified designs.

## 4. Conclusions

The Onco-Move project will provide insights into the multidimensional benefits of exercise in adults living with cancer and beyond. Using a wide spectrum of psychophysical indicators (HRQoL, sexual health, nutrition functionality, biomarkers, and genetic markers), we will compare outcomes of the structured exercise programme with those of the control group, who will not participate in such a programme. We hypothesize that the 16-week Onco-Move exercise programme will improve participants’ quality of life, sexual function, stress-response strategies, and also their physical function and body composition. We also expect to observe physiological changes related to the intervention influencing inflammation, metabolism, muscle adaptation, and cellular aging.

In particular, it will be highly relevant to understand how a Mediterranean diet profile is related to other important dimensions in PLWBC and whether participants will make any self-adjustments to their diet during the course of the project.

The Onco-Move exercise programme, once validated, will have the potential to be implemented in oncological NGOs and hospitals, as a feasible, effective and safe non-pharmaceutical aid for PLWBC.

### Dissemination of Results

All the project participants will be informed about the project results. The results will be disseminated as well among local and national cancer organisations, healthcare professionals, and policymakers. The results will be published in peer-reviewed journals. Ethical permissions have been obtained by the Bioethics Committee of the Gdansk University of Physical Education and Sport on 23 January 2025 no.1.

## Figures and Tables

**Table 1:** Eligibility criteria.

Inclusion Criteria	Exclusion Criteria
Age 18 to 55. Both genders, with no requirement for equal distribution between women and men. Currently undergoing cancer treatment or up to 5 years post-treatment. Individuals with different types of cancer. Participants who have been physically inactive and have led a sedentary lifestyle since diagnosis. Participants who commit to attending three training sessions per week. Oncologist’s approval for participation in the project. Signed informed consent from the patient to participate in the study and consent for personal data processing.	Age under 18 or over 55 at the time of registration for the project. Physically active individuals. Health conditions preventing continued participation in the study or intervention. Participants who do not commit to attending three training sessions per week. Lack of oncologist’s approval for participation in the study and training activities included in the project. Lack of signed informed consent for participation in the study and consent for personal data processing.

**Table 2:** Summary of the time-point measurements of the study outcomes.

Measurement	Baseline	8 Weeks of the Intervention	Post-Intervention (After 16 Weeks of the Intervention)	Follow-Up (3 Months After the Intervention)
**Primary outcome**				
Health-related quality of life (HRQOL) ^1^	x	x	x	x
**Secondary outcomes**				
Other quality-of-life variables ^2^	x	x	x	x
Functional fitness ^3^	x	x	x	x
Body composition ^4^	x	x	x	x
Self-reported physical fitness (IFIS)	x	x	x	x
Postural stability and balance control	x	x	x	x
Neuromuscular function	x	x	x	x
Cardiopulmonary fitness	x	x	x	x
Lower limb power and force	x	x	x	x
Grip strength	x	x	x	x
Blood lactate levels	x		x	
Biochemical markers ^5^	x		x	
Genetic analysis ^6^	x			
Nutritional assessment	x	x	x	x
Telomeres and telomerase	x		x	
Post-training assessment ^7^	x	x	x	

^1^ EORTC QLQ-C30. ^2^ Female Sexual Function Index (FSFI), International Index of Erectile Function (IIEF), UWIST Mood Adjective Checklist (UMACL), The Feeling of Stress Questionnaire (KPS), Four-Item Patient Health Questionnaire For Anxiety And Depression (PHQ-4), NEO Five-Factor Inventory (NEO-FFI), and State–Trait Anxiety Inventory (STAI). ^3^ Senior Fitness Test (SFT), Short Physical Performance Battery (SPPB). ^4^ Inbody 720: body fat (%), fat mass (kg), fat-free mass (kg), bone mass (kg), body mass index (BMI; kg/m2), intra-extracellular water (L), waist-to-hip ratio (WHR). ^5^ IL-6 (Interleukin-6), IL-15 (Interleukin-15), Irisin, BDNF (Brain-Derived Neurotrophic Factor), SPARC (Secreted Protein, Acidic, and Rich in Cysteine), Decorin. ^6^ CE, ACTN3, PPARGC1A, BDNF, VEGF, IL6, NOS3, HIF1A, TNF-α, mTOR pathway, telomeres, and telomerase. ^7^ Online post-training assessment forms filled out by the participants up to 24 h after each training session.

## Data Availability

The original contributions presented in this study are included in the article/Supplementary Materials. Further inquiries can be directed to the corresponding author.

## Supplementary Materials

The following supporting information can be downloaded at: https://www.mdpi.com/article/10.3390/nu18010100/s1, Table S1: CONSORT 2025 checklist; Table S2: Description of the Onco-Move exercise programme; Table S3: Exercise equipment for the Onco-Move project.

## Abbreviations

The following abbreviations are used in this manuscript:

PLWBC	People living with and beyond cancer
NGO	Nongovernmental organization
ACS	American Cancer Society
ACSM	American College of Sports Medicine
WHO	World Health Organization
HRQoL	Health-related quality of life
RCT	Randomized controlled trial
CERT	Consensus on Exercise Reporting Template
GUPES	Gdansk University of Physical Education and Sport
QoL	Quality of life
FSFI	Female Sexual Function Index
IIEF	International Index of Erectile Function
UMACL	UWIST Mood Adjective Checklist
KPS	The Feeling of Stress Questionnaire
PHQ-9	Patient Health Questionnaire For Anxiety And Depression
NEO-FFI	NEO Five-Factor Inventory
STAI	State–Trait Anxiety Inventory
IFIS	Self-reported physical fitness
IPAO	Physical activity objectives
PA	Physical activity
SFT	Senior Fitness Test
SPPB	Short Physical Performance Battery
10MWT	10-Metre fast walk test
MEDAS	Mediterranean Diet Adherence Screener
EV	Evaluation
DV	Development
TS	Transitional stimulus
ITT	Intention-to-treat principle
MMRM	Mixed-effects model for repeated measures
