# Supplementary material for: Supervised 16-Week Multicomponent Exercise Training Programme for 18–55-Year-Old People Living with and Beyond Cancer—CONSORT 2025-Based Study Protocol of the Pilot Onco-Move Randomized Controlled Trial
Source: Nutrients. 2025 Dec 27;18(1):100. doi: 10.3390/nu18010100 (PMC12787456; doi:10.3390/nu18010100)
Supplement: Supplementary file 1 [file nutrients-18-00100-s001.zip › Table S2_Onco Move exercise programme.pdf]

Table S2. Description of The Onco-Move Exercises Programme.

Name of the exercise	Exercise description
Stability Squat on Sensory Disc	**Initial position:** Stand tall on the disc(s), feet hip-width apart, arms in front for balance, core gently braced.<br>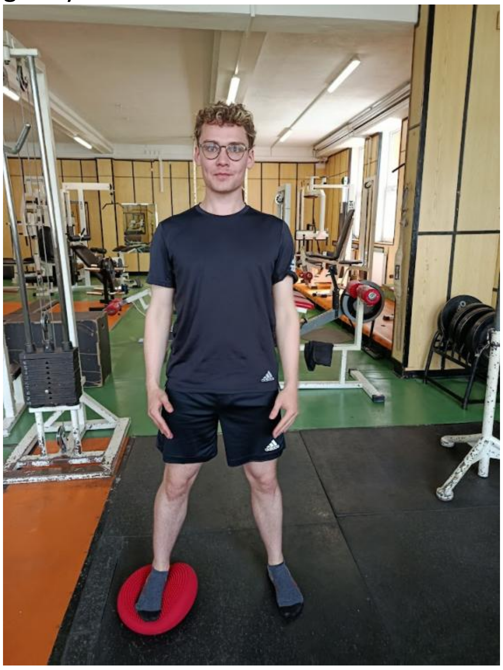<br>**Exercise:** Squat while standing on balance disc to activate stabilizers<br>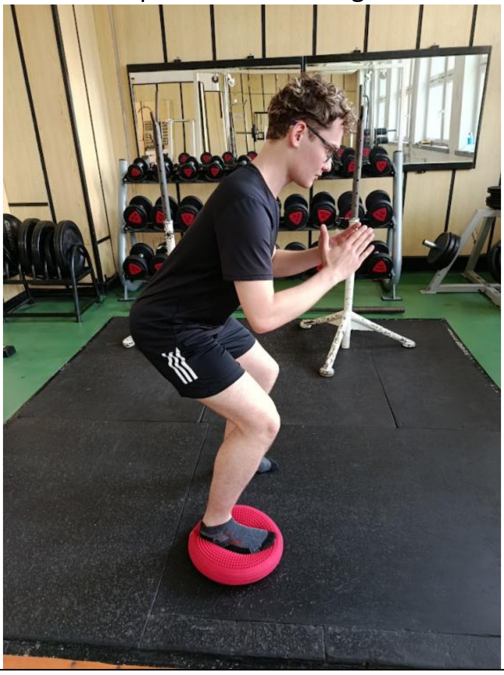

Mini Band Lateral Steps	**Initial position:** Mini band above knees, slight squat position (athletic stance), feet hip-width, chest lifted, knees soft.<br>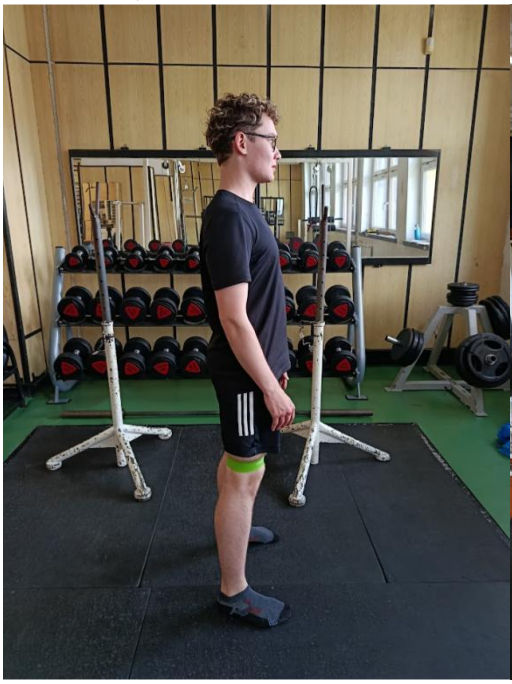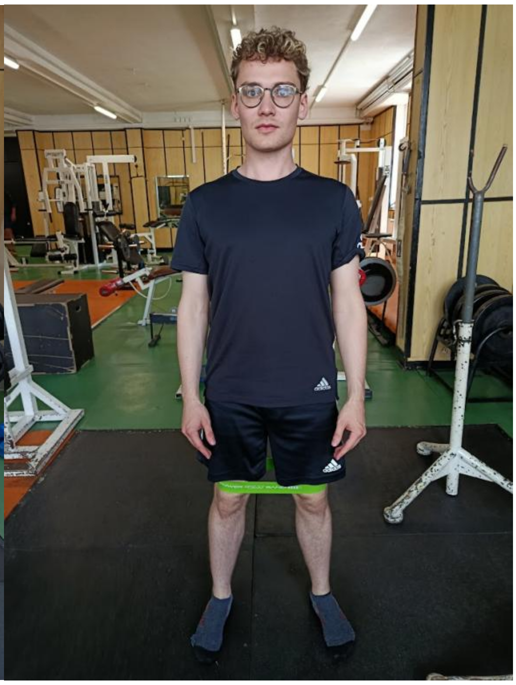<br>**Exercise:** Step side-to-side against band resistance<br>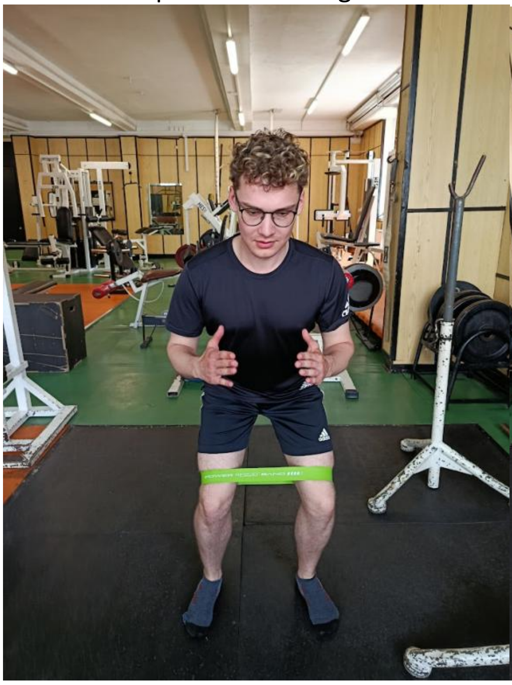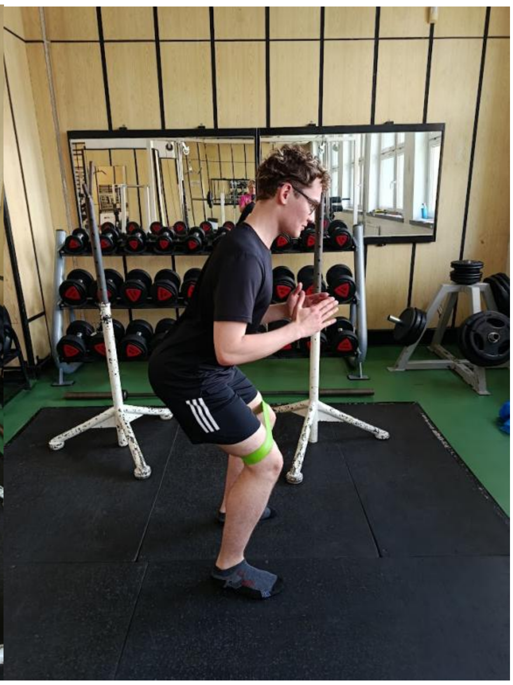

Hip Thrust with mini band	**Initial position:** Upper back on the floor, feet flat, knees bent, mini band around knees, arms stabilizing weight, core braced.<br>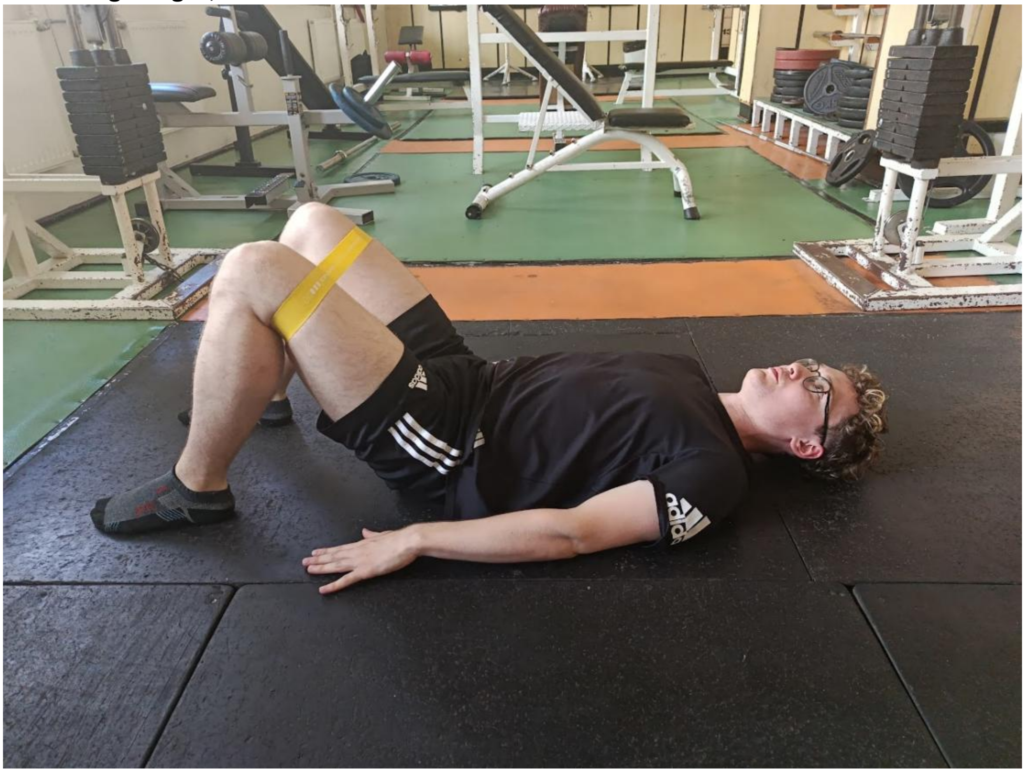<br><br>**Exercise:** Bridge hips up with mini band around knees, pause at top, bring your knees to the side.<br>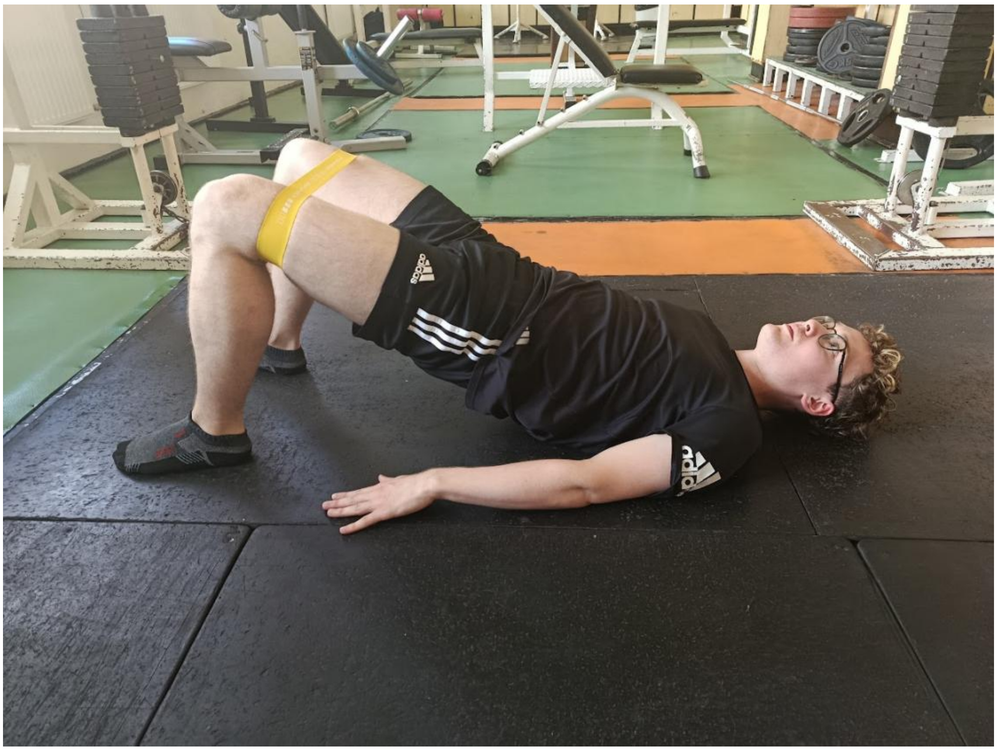

Overhead Dumbbell Press	**Initial position:** Standing, dumbbells at shoulder height, elbows under wrists, palms facing forward or in, spine neutral.<br>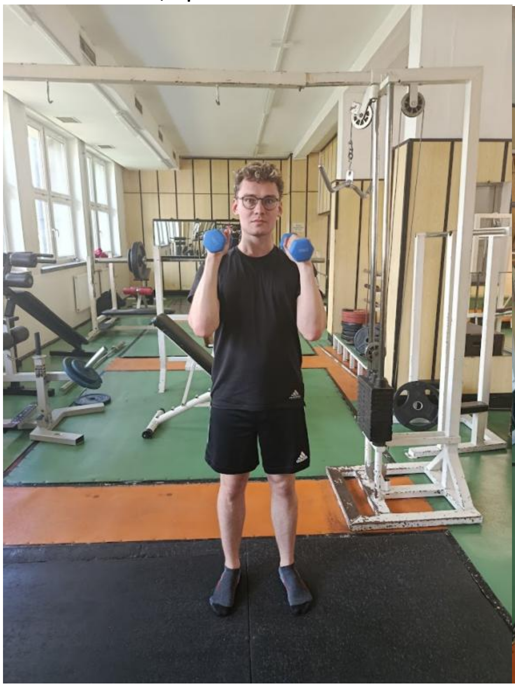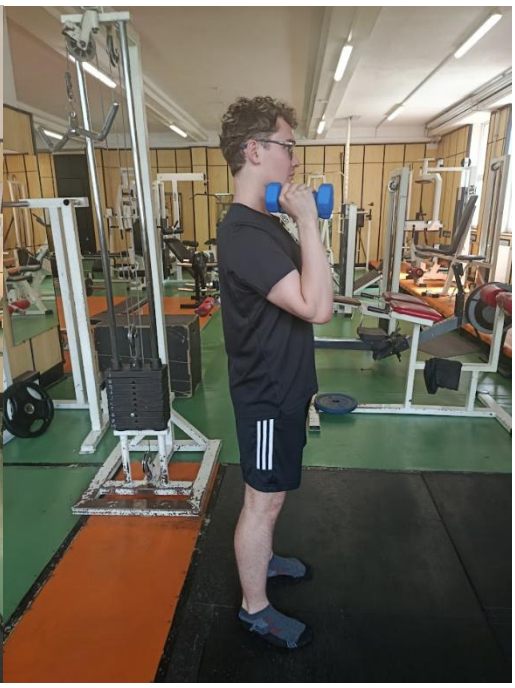<br>**Exercise:** Push weights overhead in a controlled motion<br>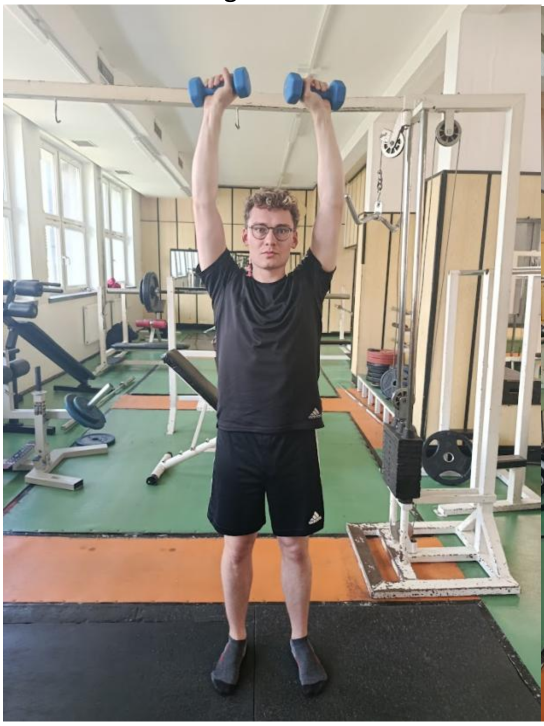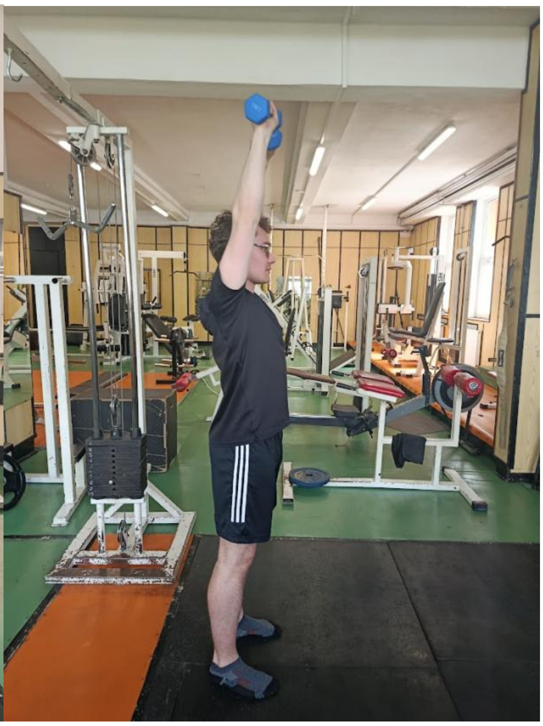

March with Arm Band Raise	**Initial position:** Stand tall with flat band in hands at chest level, elbows soft, feet hip-width, core engaged. 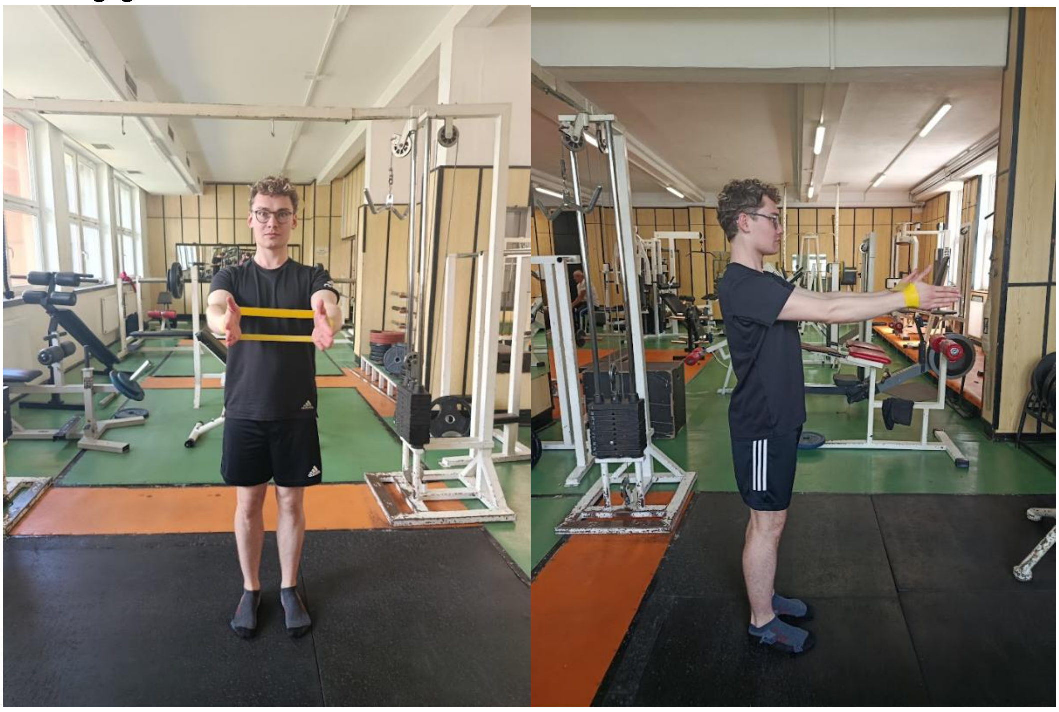 **Exercise:** March in place while pulling band overhead

Standing one-arm overhead dumbbell triceps extension	**Initial position:** Stand upright with your feet shoulder-width apart. Hold a dumbbell in one hand. Your arm is bent, and the dumbbell is at head height.<br>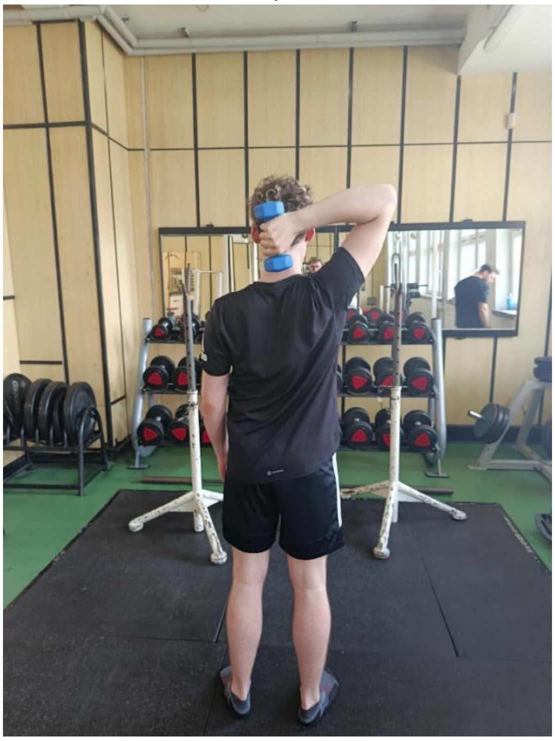<br>**Exercise:** Raise the dumbbell overhead, keeping your elbow close to your head and slightly bent.<br>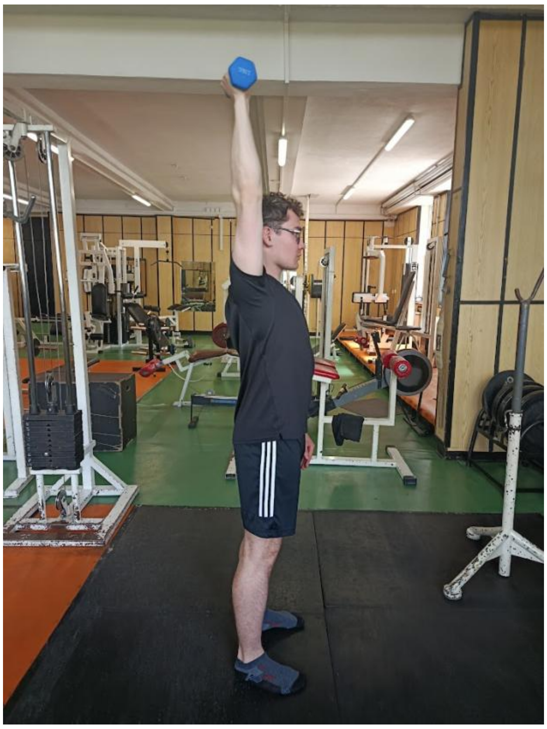

	**Exercise modification A:**<br>Hold a mini band with both hands. Place your right hand on your left shoulder – this hand will provide resistance and stability. Position your right hand close to your body at mid-chest level. The elbow should be fully bent, and the upper arm kept vertical and tight to your torso.<br><br>**Exercise:** Begin the movement by extending your left elbow, pushing the band down across your chest diagonally. Continue the motion until your arm is fully extended, focusing on contracting the triceps. Pause briefly at the bottom of the movement while maintaining tension. Slowly return to the starting position in a controlled manner, keeping resistance on the band throughout the range of motion.<br>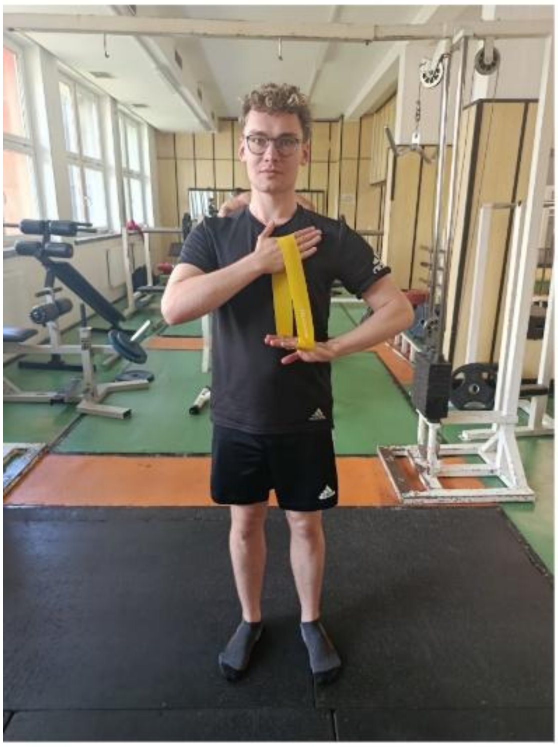 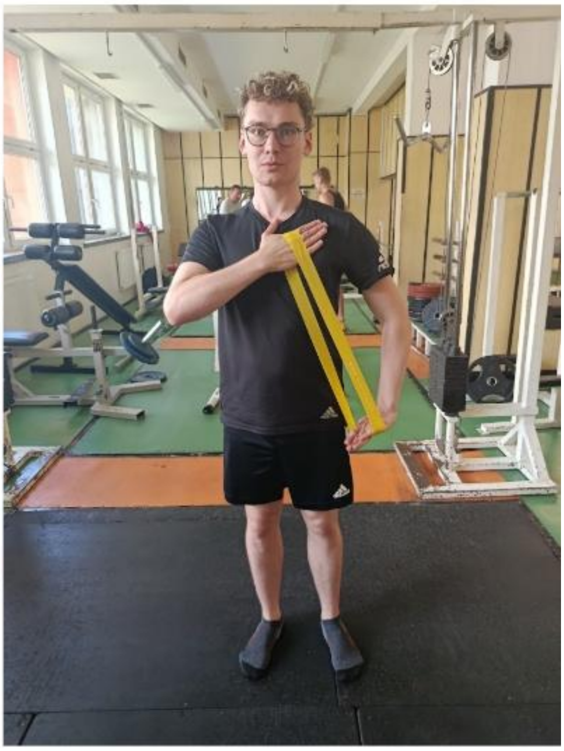

Biceps Curl with Dumbbells	**Initial position:** Stand, arms extended down, palms forward, elbows close to torso, dumbbells in hands.<br>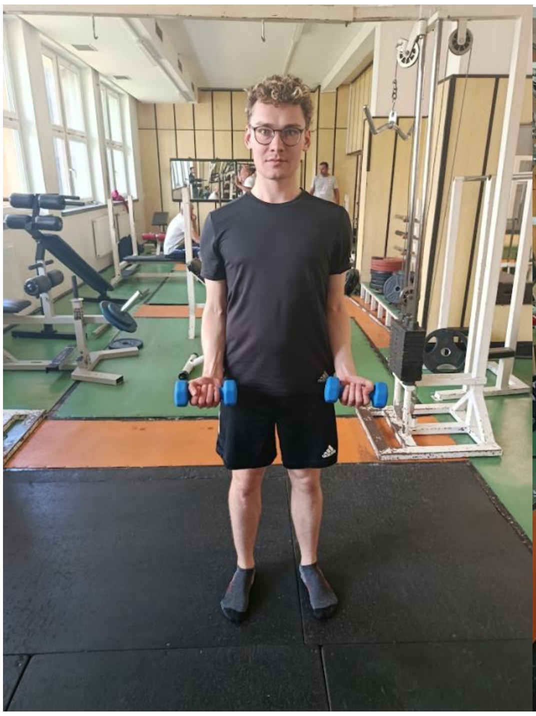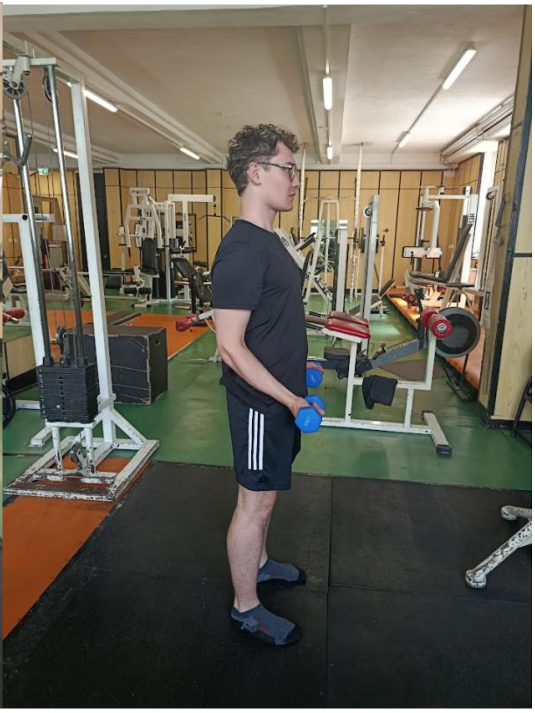<br>**Exercise:** Curl with control, elbows tight to body<br>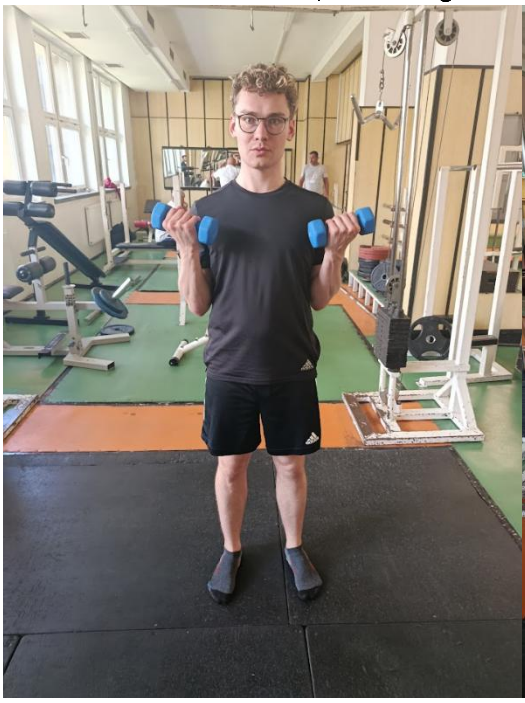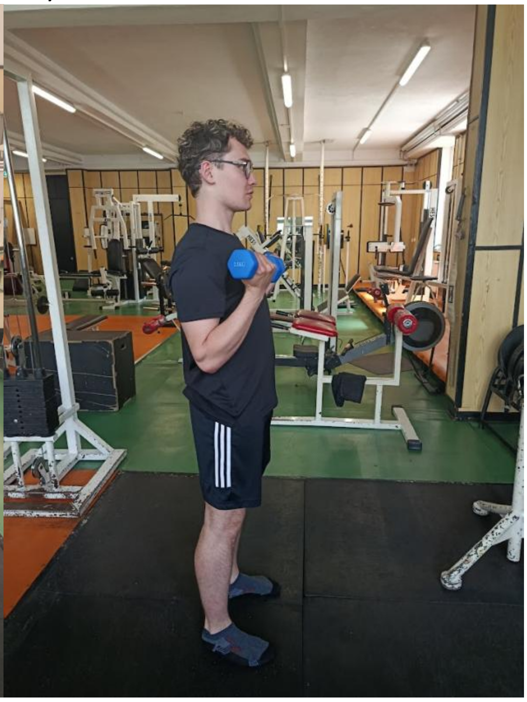

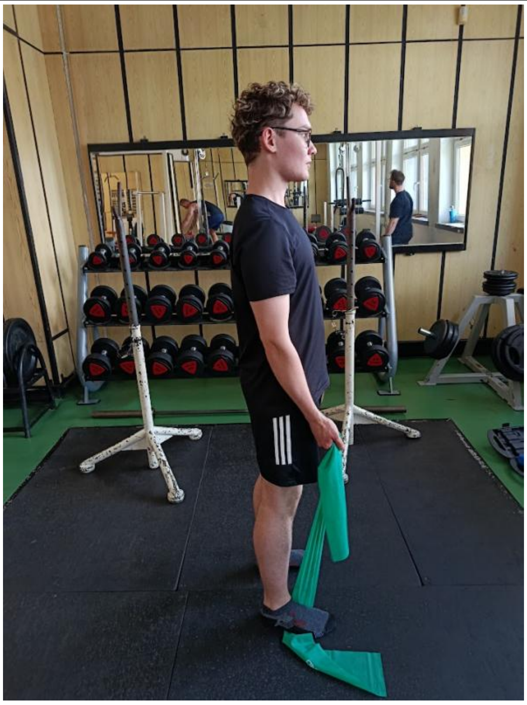
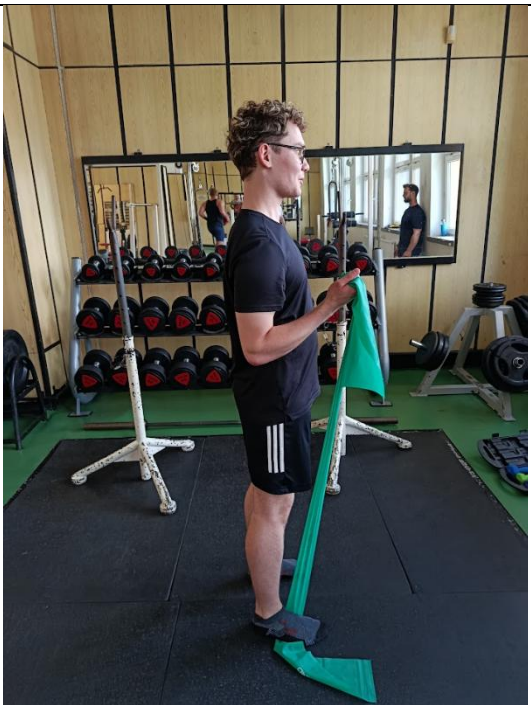

Exercise modification A: Stand on the center of a resistance band with your feet hip-width apart. Hold one end of the band in one hand, palm facing forward. Keep your arm fully extended by your side. Maintain a straight back, chest lifted, and shoulders relaxed. Engage your core for stability.

Exercise: Begin by slowly curling your hand upward by bending at the elbow, keeping your upper arm stationary. Exhale as you lift, bringing your hand toward your shoulder. At the top of the movement, squeeze your bicep and hold for a brief pause.
Then, inhale as you slowly lower your hand back down to the starting position in a controlled motion.

Lunge with Torso Twist with Pilates Ball Support	**Initial position:** Stand straight with your feet hip-width apart, arms extended in front of you at chest height (holding the ball), prepare to step back.<br>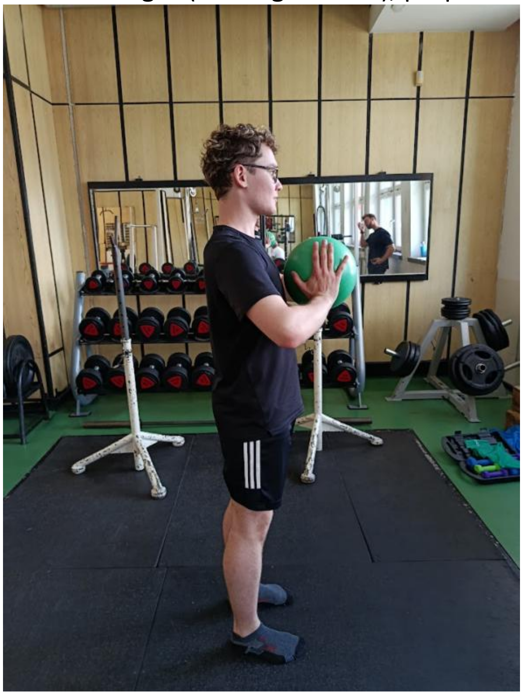<br>**Exercise**: Step into lunge, gently rotate torso toward front leg, exhale and squeeze the ball<br>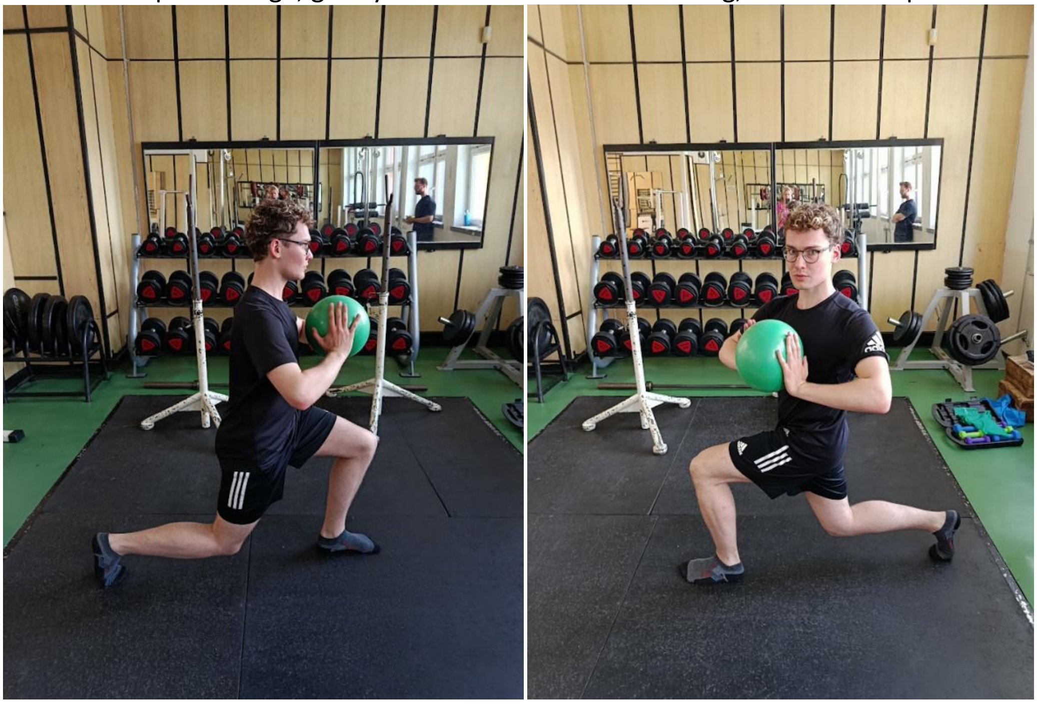

Crunch with Pilates Ball Support	**Initial position:** Lie on mat with knees bent, feet flat, ball between knees, hands crossed over chest.<br>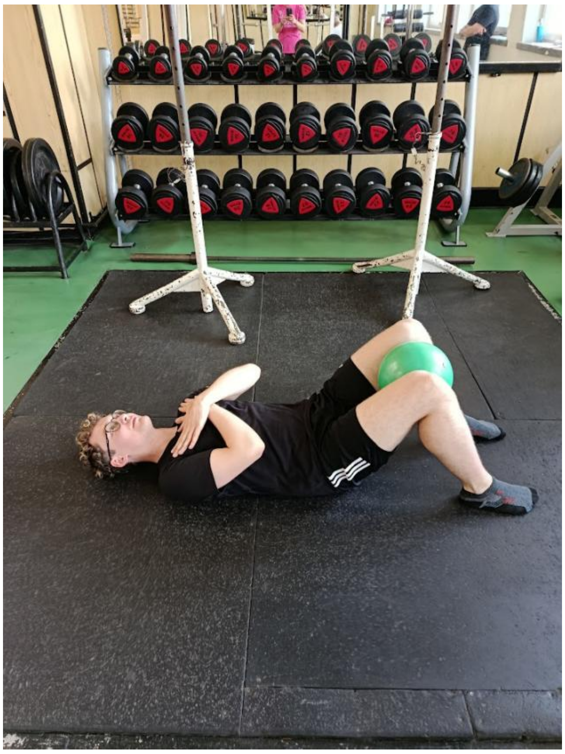<br>**Exercise:** Crunch with ball behind back or knees, small controlled lift<br>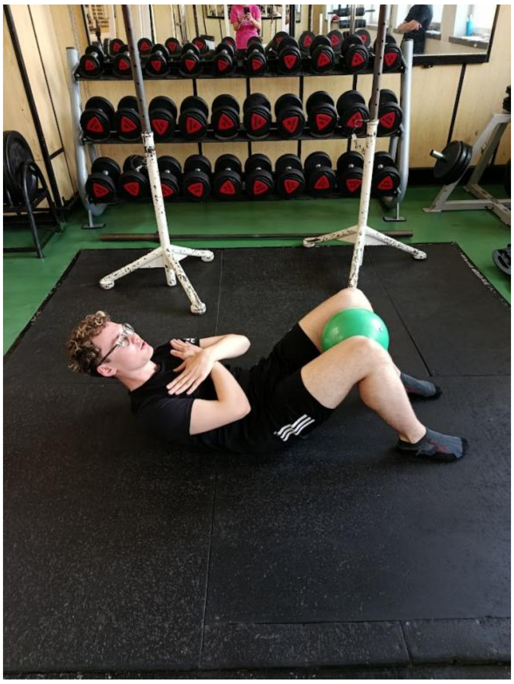

	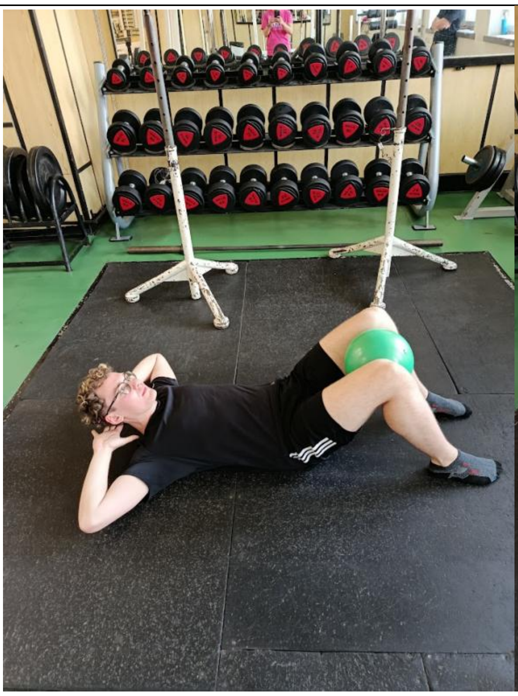 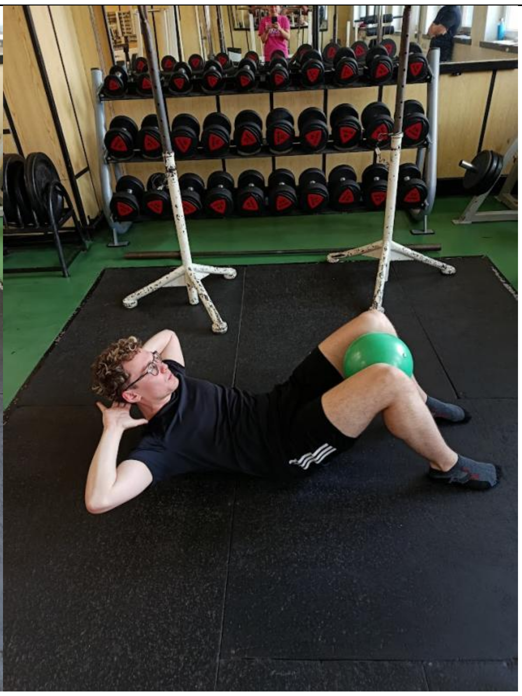 **Exercise modification:** Lie on mat with knees bent, feet flat, ball between knees, hands placed behind the head. Bend your torso, keeping your elbows wide and your chest open.

<table>
<tr>
<td>Lat pulldown wide grip</td>
<td>Initial position: Sit facing the lat pulldown machine with your knees secured under the rollers, and grasp the bar with a wide overhand grip, arms fully extended overhead.
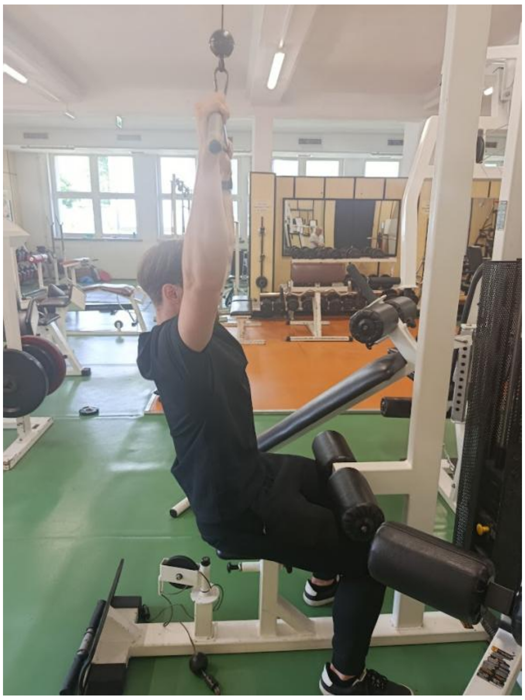
Exercise: Inhale as you lean back slightly and puff out your chest; initiate the movement by pulling your shoulder blades down and together, then pull the bar toward your upper chest while driving your elbows down and in toward your sides—pause briefly at peak contraction, then exhale as you smoothly return the bar to the starting position.
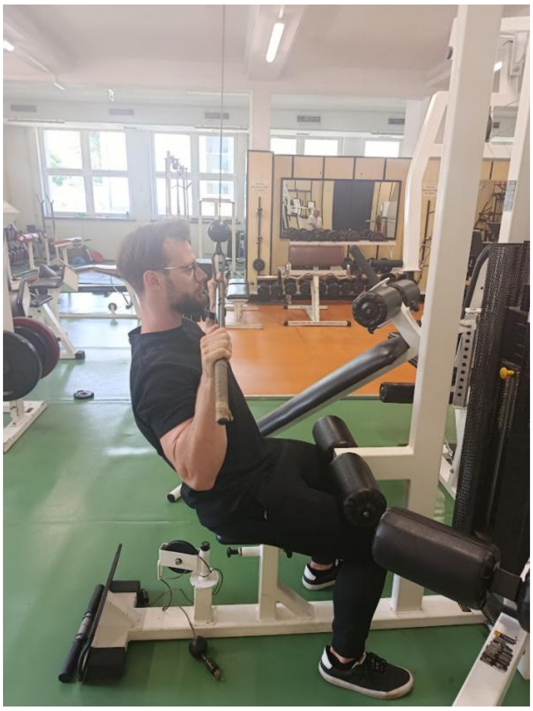</td>
</tr>
</table>

Exercise modification A: Lie on your back with knees bent and feet flat on the floor.
Extend both arms overhead and hold a dumbbell vertically with both hands above your head. Slightly lift the dumbbell off the floor, keeping your arms extended but not locked, and engage your core for stability.

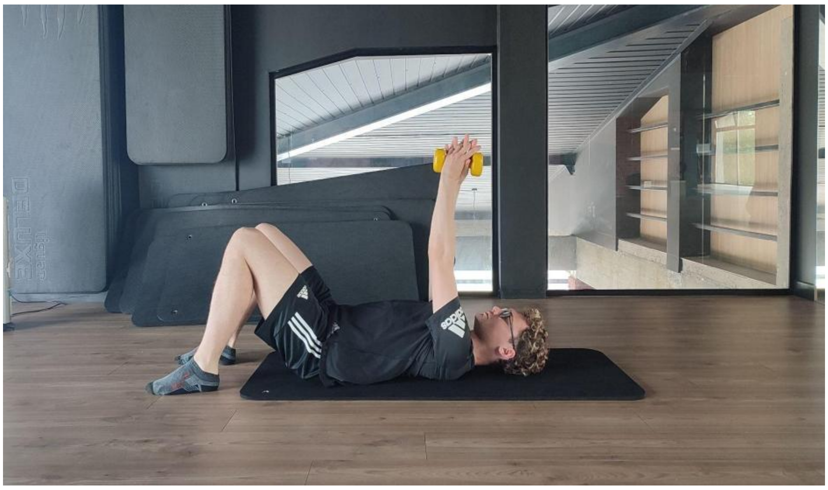

Exercise: Exhale as you lift and pull the dumbbell from the floor to directly above your chest, keeping your arms extended with a slight bend in the elbows.
Then, inhale as you reverse the movement, drawing the dumbbell upward and backward over your head. Lower it in a controlled motion beyond your head to return to the starting position, maintaining a slight bend in the elbows throughout.

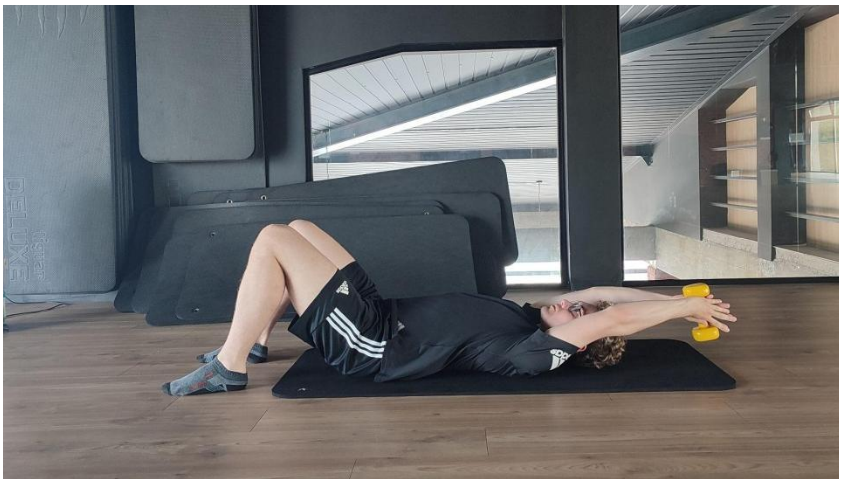

Box squat / assisted box squat	**Initial position:** Stand in front of a box or bench, feet shoulder-width apart, toes slightly turned out, core engaged, arms forward for balance.<br>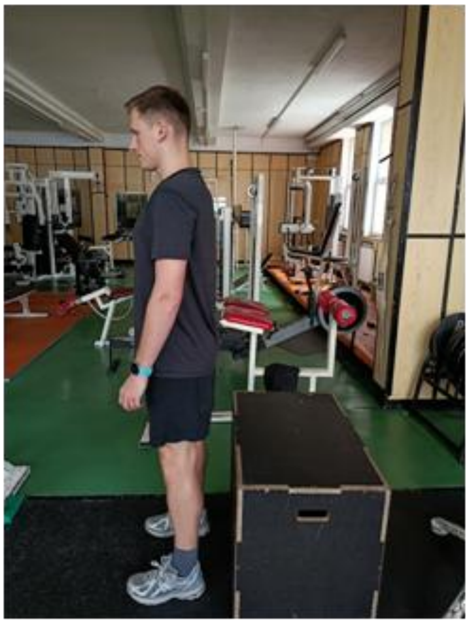<br>**Exercise:**<br>**Variant A**<br>Lower hips back and down to sit briefly on the box, then drive through heels to return to standing. Focus on control and alignment<br>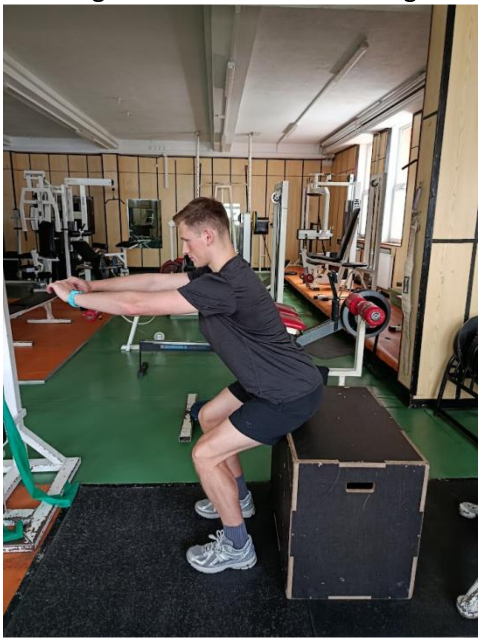

Variant B
Initial position: Stand in front of a box or bench, feet shoulder-width apart, toes slightly turned out, core engaged, plate hold in front of your chest
Exercise: Lower hips back and down to sit briefly on the box, then drive through heels to return to standing. Keep the plate at chest level while performing.

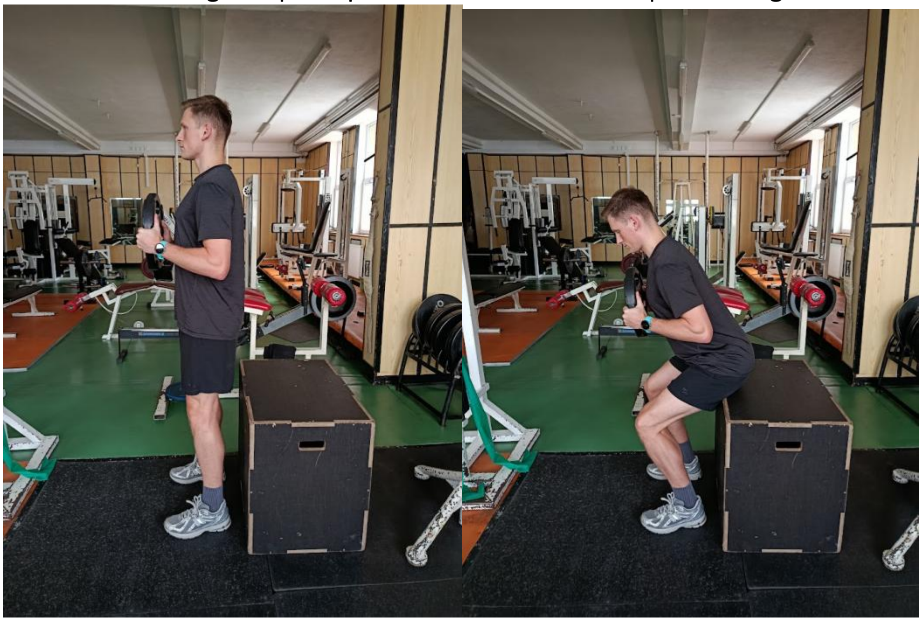

Exercise modification:
Use a dowel or resistance band for support; perform next to a stable surface for assistance with balance.

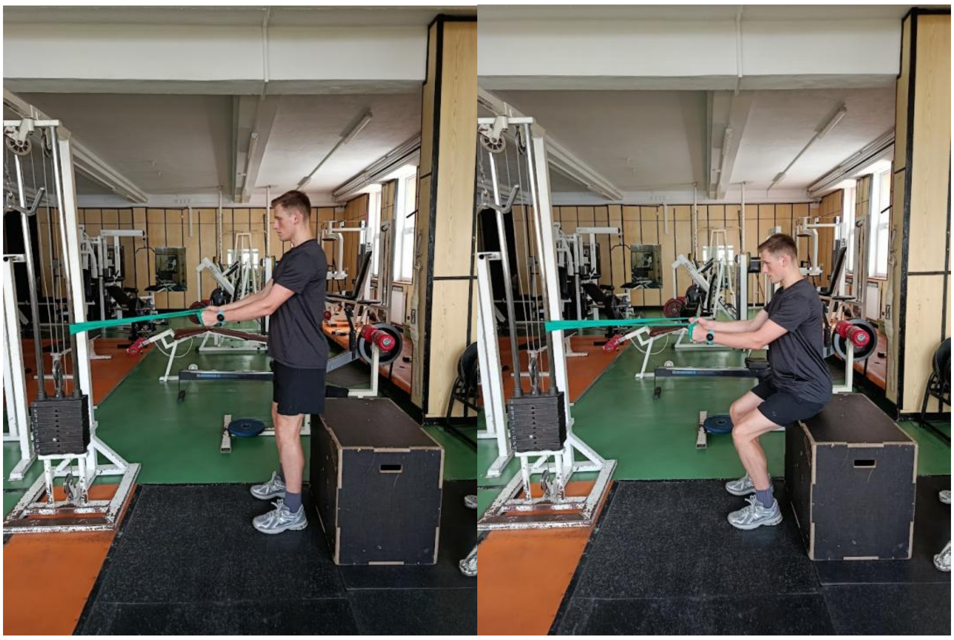

Bent over row	**Initial position:** Stand with feet hip-width apart, hold weights in hands, hinge at hips to bring torso forward (back flat, core engaged), arms extended down. 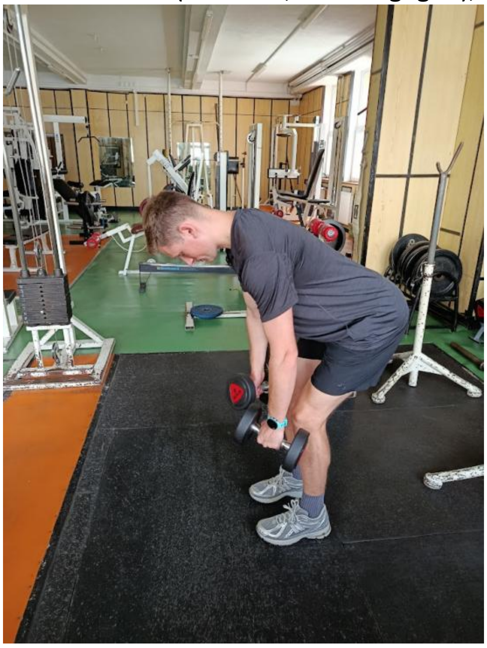 **Exercise:** Pull elbows back, bringing weights toward the torso, squeezing shoulder blades together. Lower with control. 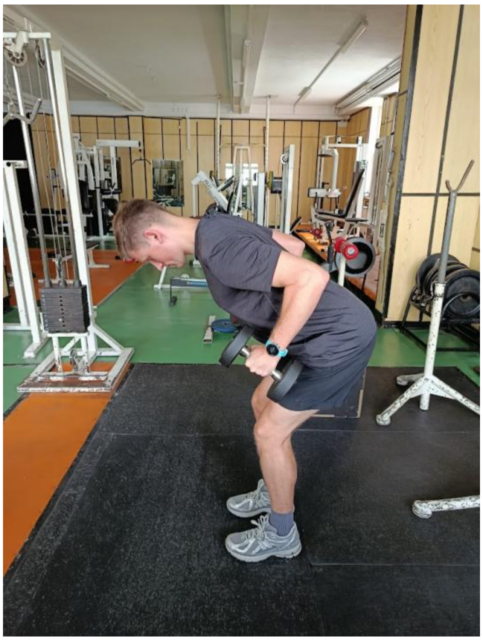

	**Exercise modification:** Perform seated with resistance band anchored under feet; use lighter weights or support chest on an incline bench for reduced strain.<br>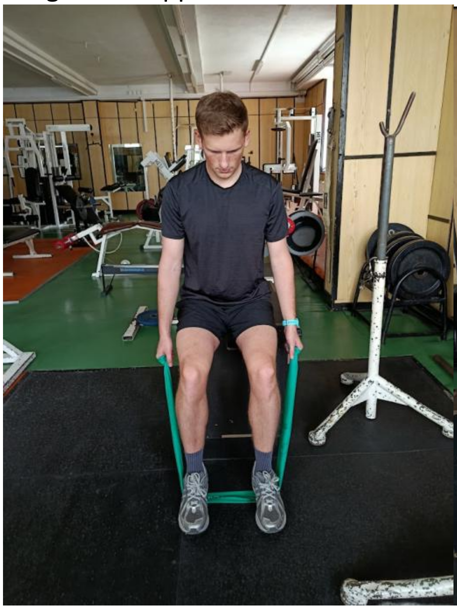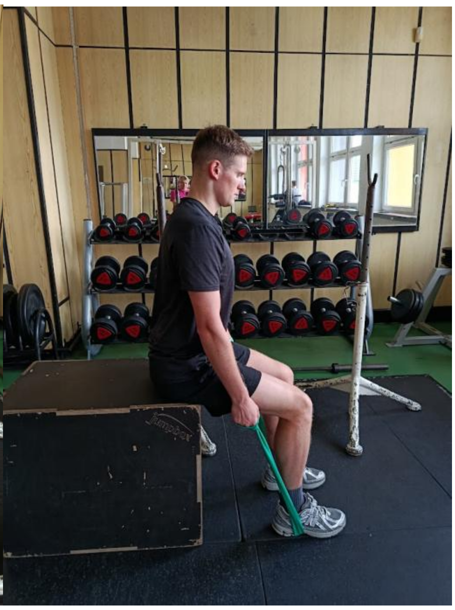<br>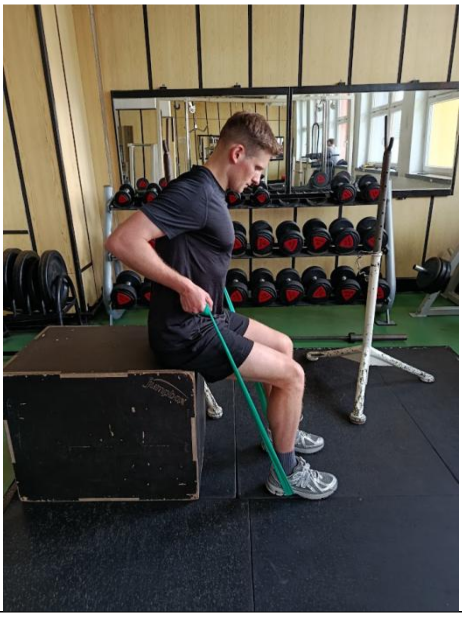

Glute bridge	**Initial position:** Lie on your back, knees bent, feet flat on the floor hip-width apart, arms at sides, core gently engaged.<br>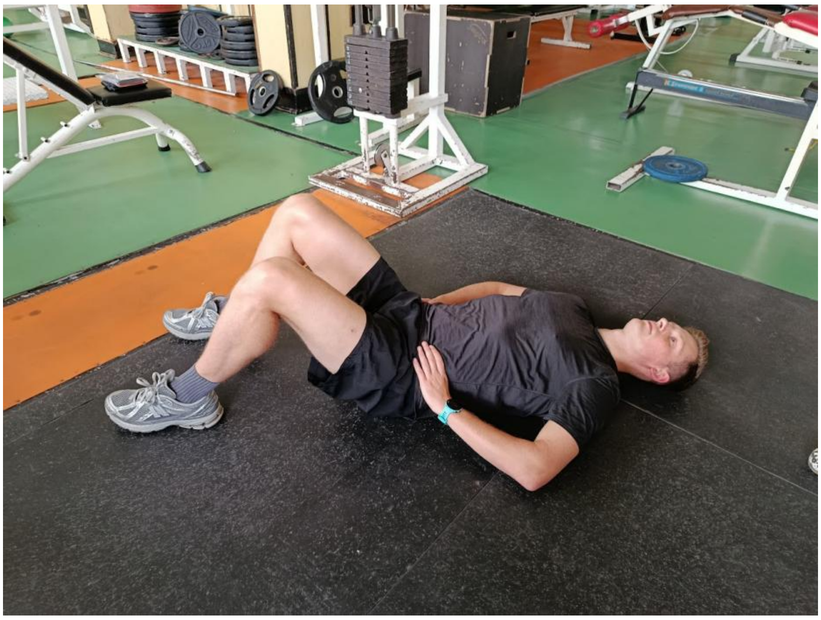<br>**Exercise:**<br>**Variant A**<br>Press through heels to lift hips toward the ceiling, squeezing glutes at the top. Lower slowly without touching the floor.<br>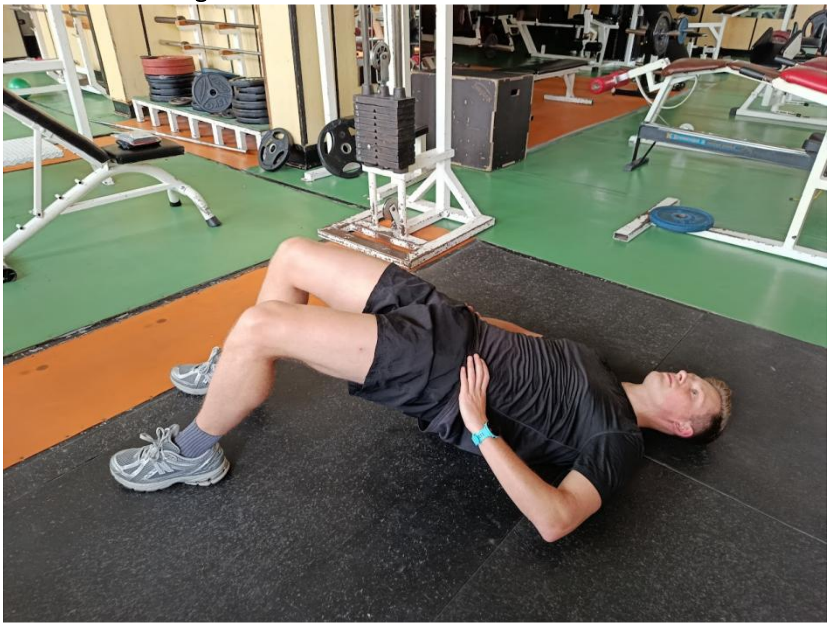

Variant B
Initial position: Lie on your back, knees bent, feet flat on the floor hip-width apart, arms hold the weight on the hips.
Exercise: Press through heels to lift hips toward the ceiling, squeezing glutes at the top. Lower slowly without touching the floor. Maintain the weight at hip level during the movement.

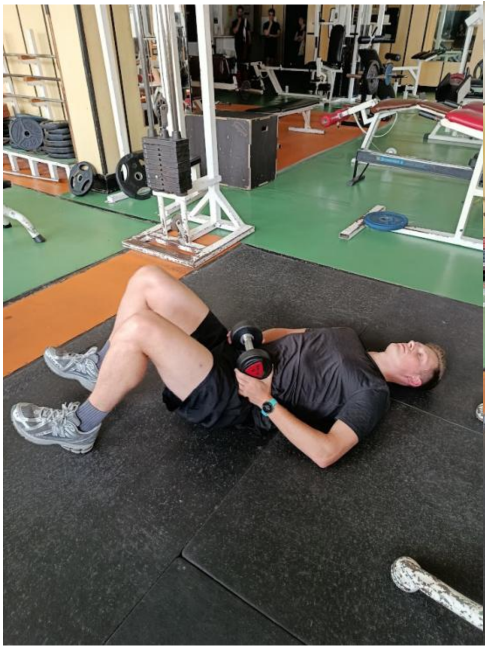
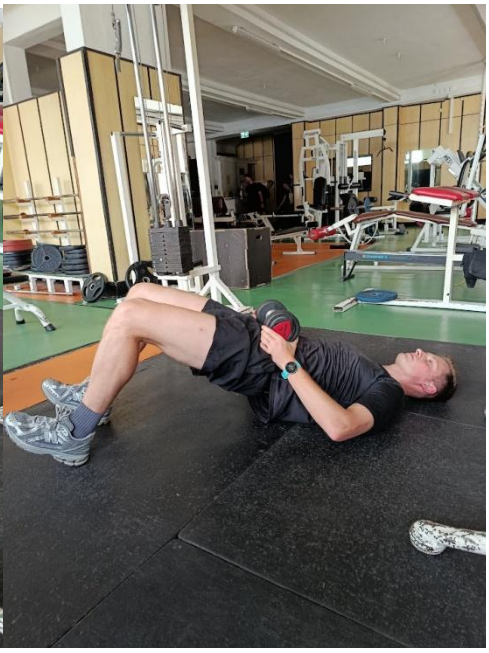

Exercise modification:
perform with feet elevated on a low surface for easier activation.

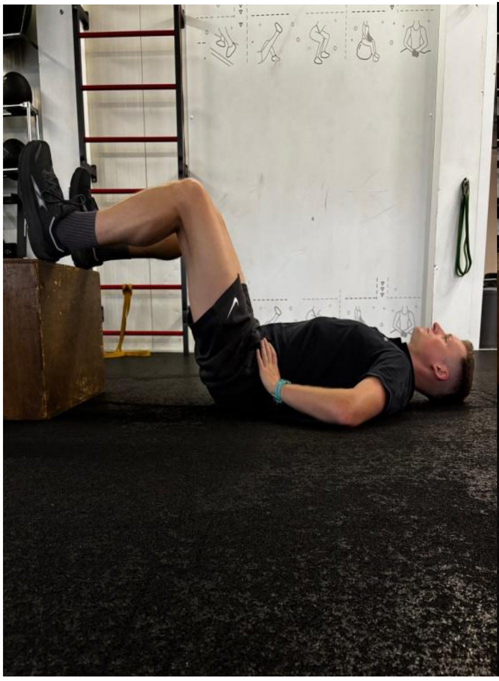
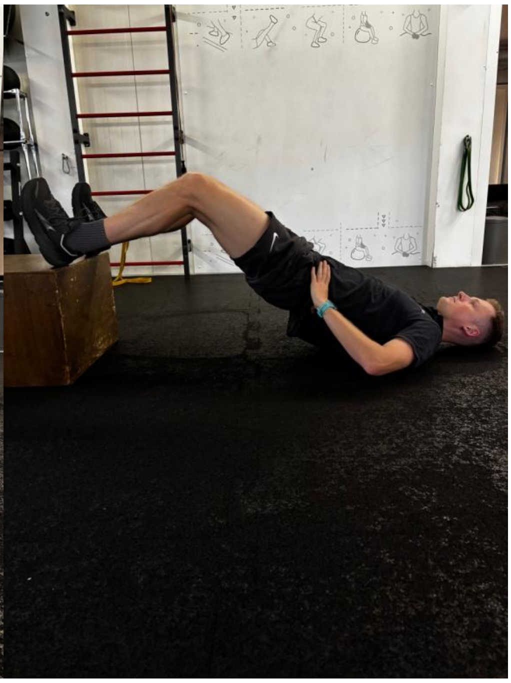

Push - ups	**Initial position:** Start in a high plank position, hands slightly wider than shoulders, body in a straight line, core and glutes engaged. 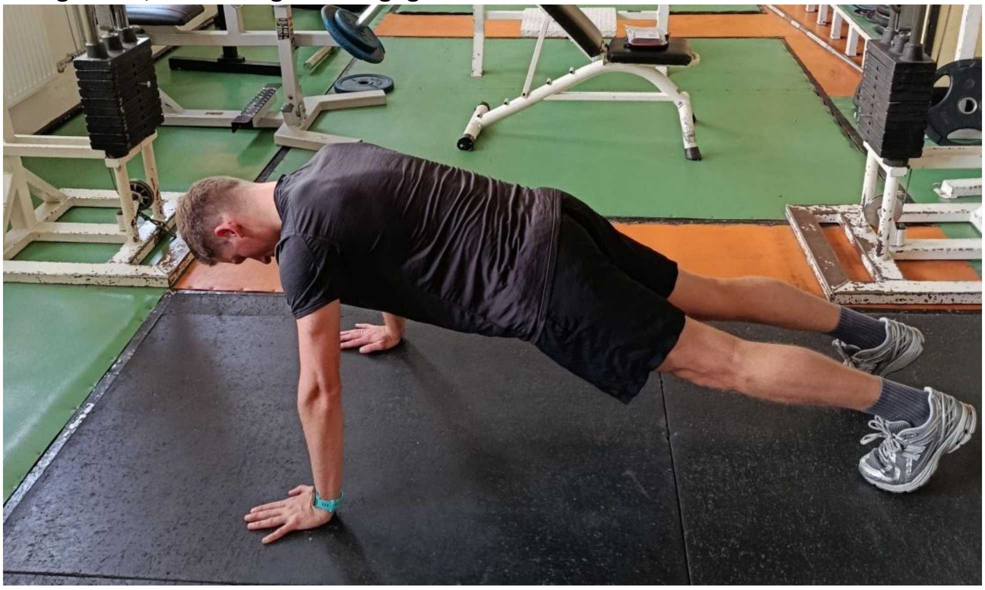 **Exercise:** Lower chest toward the floor by bending elbows, then push back up to the starting position. Maintain full-body tension throughout. 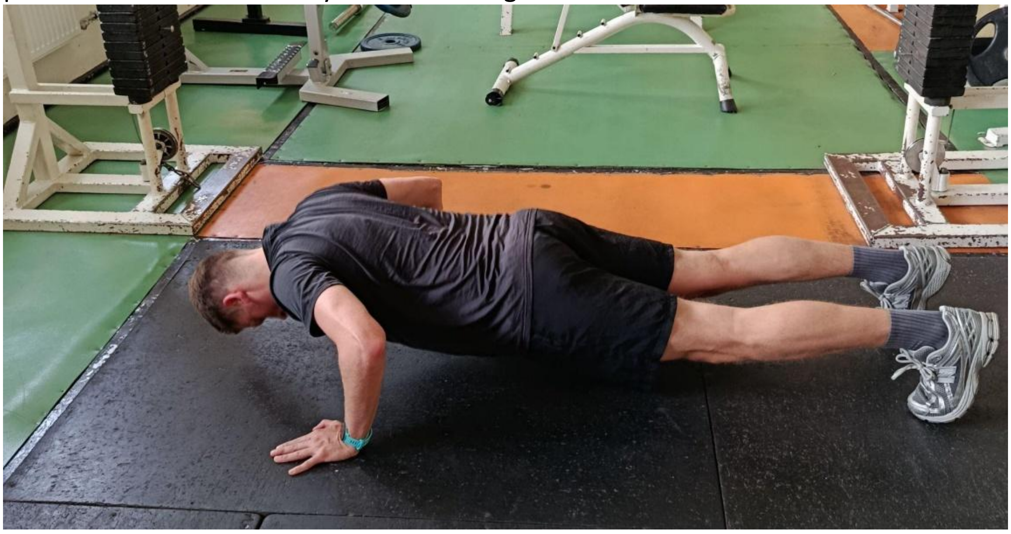

	**Exercise modification:**<br>Perform on knees or incline (e.g., hands on a bench or box) to reduce load.<br>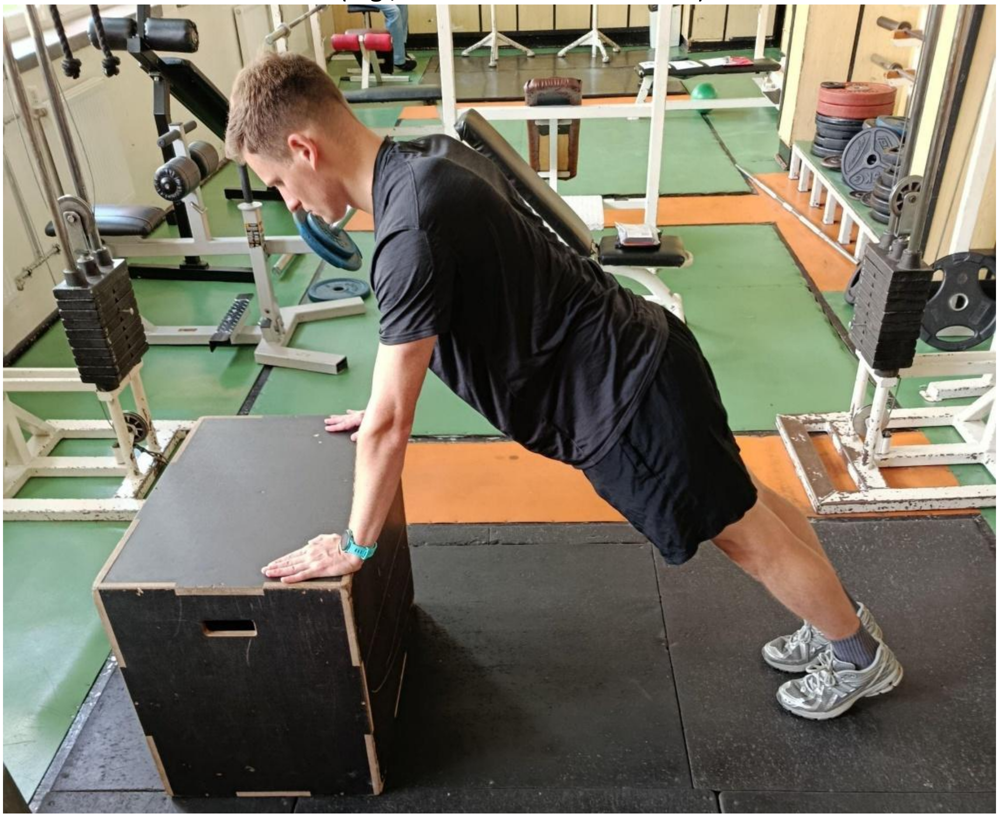<br>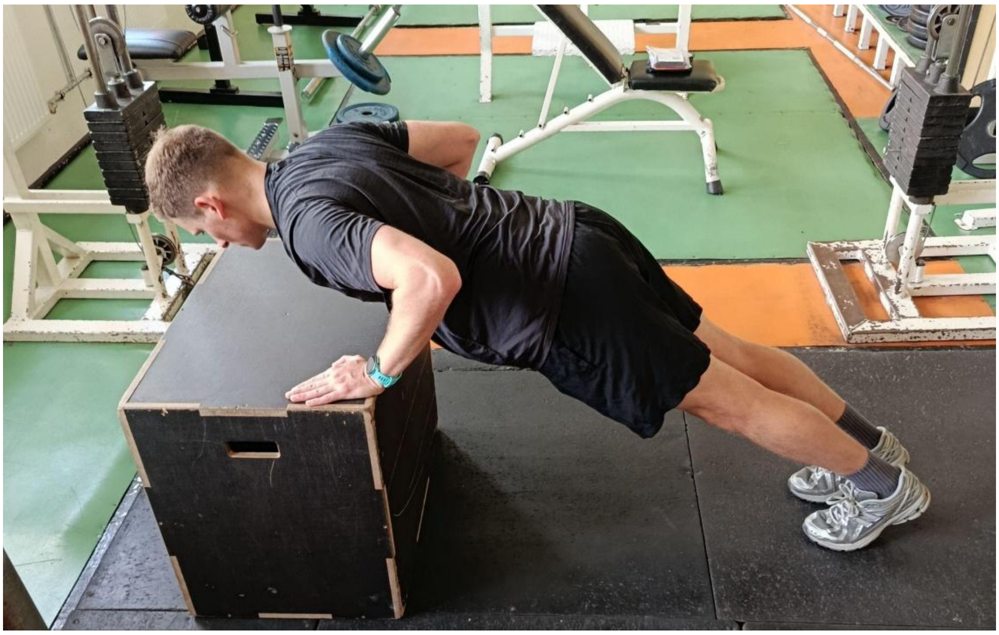

Plank	**Initial position:** Forearms on the ground, elbows under shoulders, drop knees to the ground while keeping a straight line from head to knees, core braced.<br>**Exercise:** Hold the position without letting hips sag or rise. Focus on maintaining steady breathing and full-body tension.<br>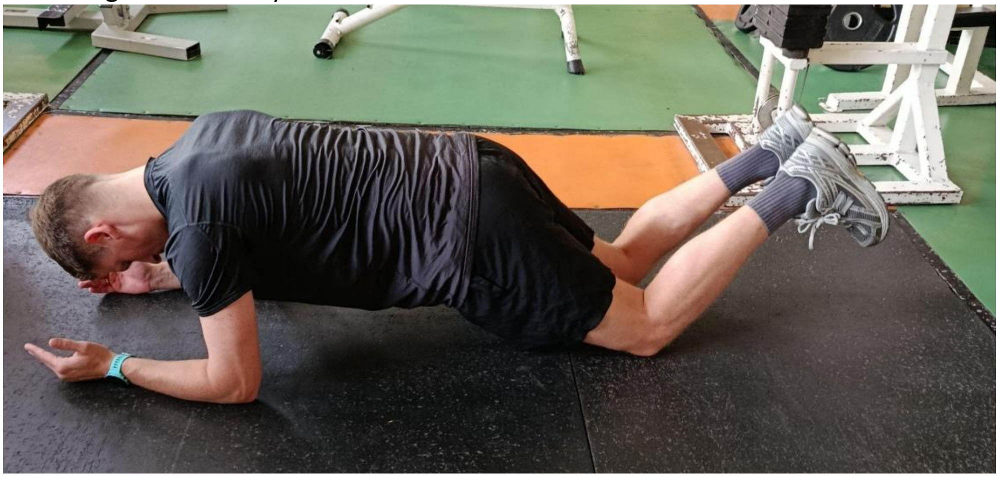

Lunge	**Initial position:** Stand tall, feet hip-width apart, hands on hips or at sides, core engaged.<br>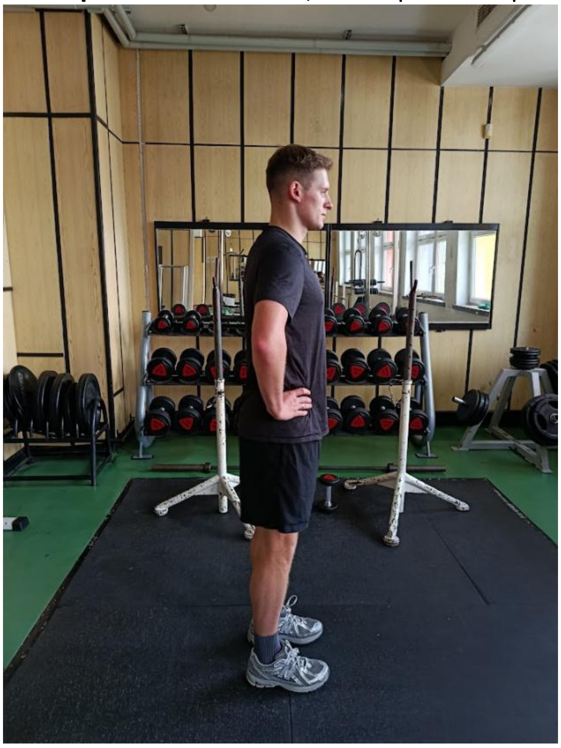<br>**Exercise:** Step forward with one leg and lower hips until both knees are at 90 degrees. Push back to the starting position and alternate legs.<br>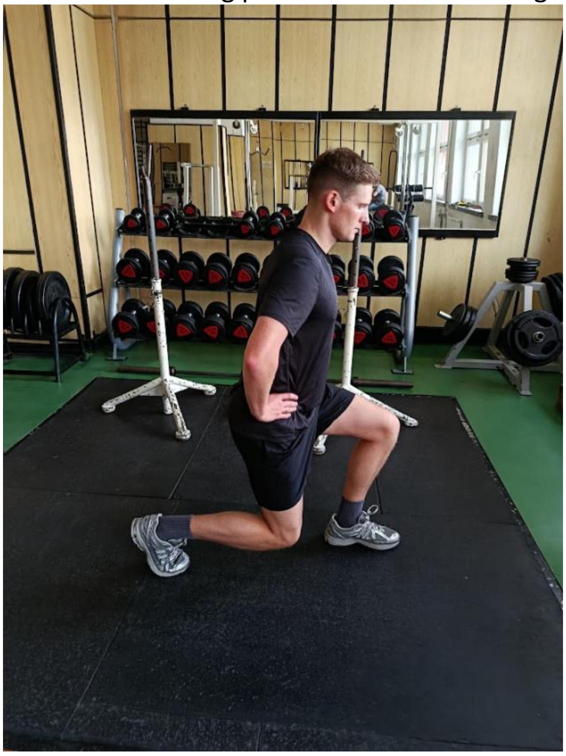

	**Exercise modification:**<br>Hold a dumbbell in each hand to add weight maintaining initial position. Step forward with one leg and lower hips until both knees are at 90 degrees. Push back to the starting position and alternate legs.<br>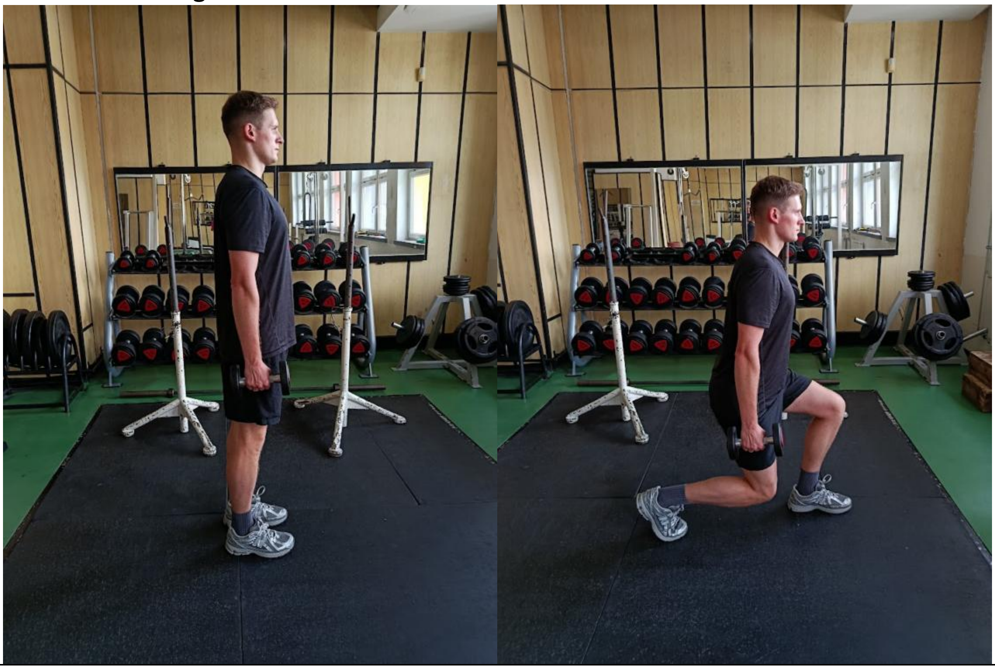

Interval cycling	**Initial position:**<br>Exercise is performed on a cycle ergometer in a seated position. The saddle height is individually adjusted so that the participant’s knee remains slightly flexed (approximately 25–30°) at the bottom of the pedal stroke. The handlebars are positioned to allow a comfortable grip without excessive forward lean, ensuring proper posture and minimizing upper body strain. Foot straps are used to secure the feet to the pedals to enhance stability and pedaling efficiency.<br><br>**Exercise:**<br>The interval training consists of alternating periods of high-intensity cycling and active recovery, performed according to the predefined protocol parameters, under supervision and standardized conditions.<br><br>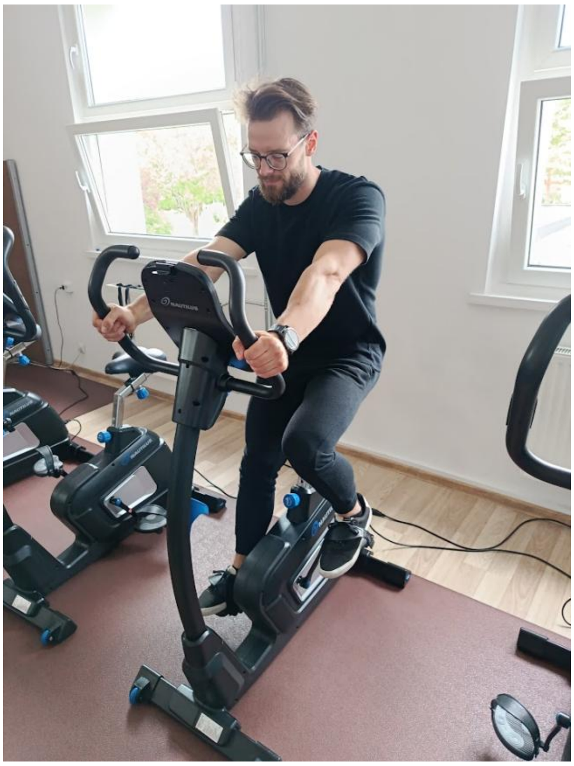

90 degree back extensions	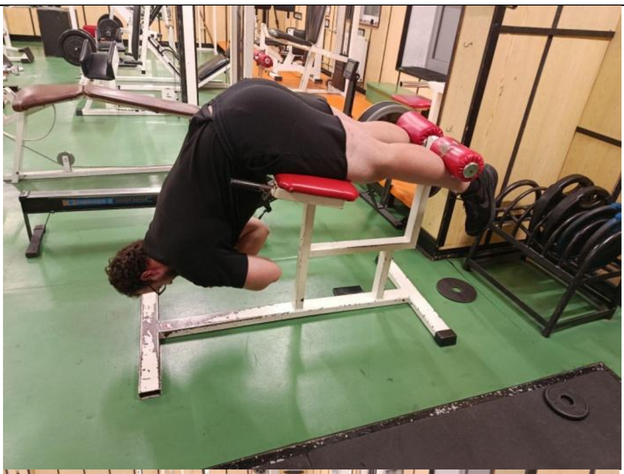<br>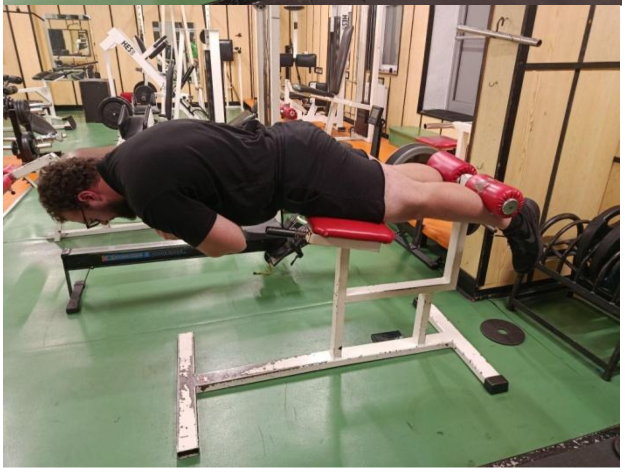<br><br>**Initial position:**<br>Get on the machine by resting your hips on the pad and anchoring your legs underneath the attachment behind the pad. The point of contact with the back attachment should be the lower calf or the achilles tendon. The point of contact with the pad should be the hips or upper thigh area. Suspend your upper body facing downward by generating upward force with your legs against the back attachment.<br><br>**Exercise:**<br>Keeping your knees and lower back straight, initiate hip flexion. Try to descend your upper body downward until it is perpendicular to the floor, or to the maximum of one's mobility and comfort. After reaching the desired end range, initiate hip extension until returned to the starting position.<br><br>**Exercise modifications:**<br>If unable to perform hip flexion due to mechanical disadvantage or other limitations, perform weighted isometric holds in full hip extension.

Dumbbell romanian deadlift	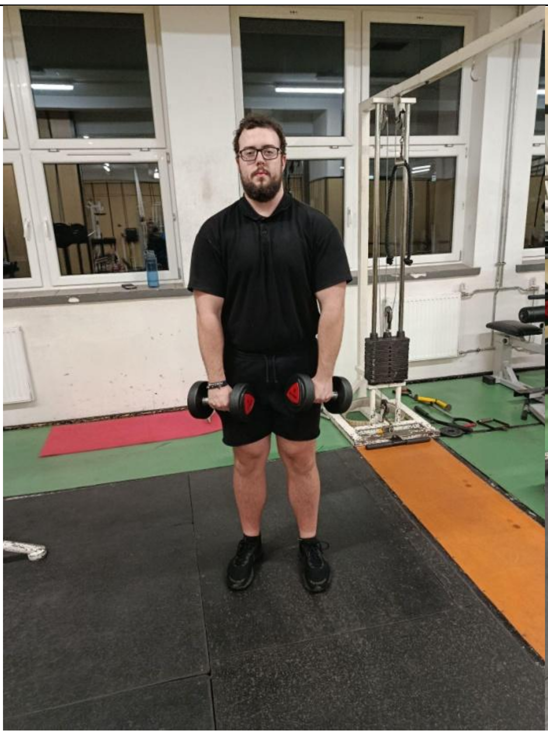 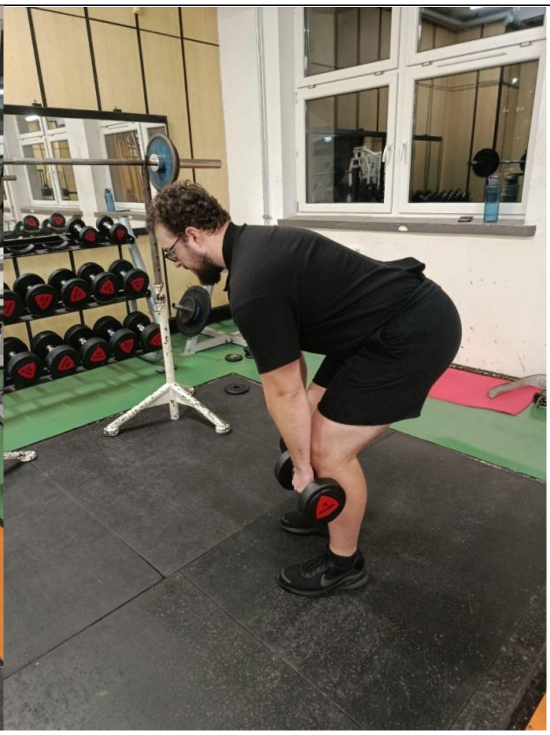<br><br>**Initial position:**<br>Stand upright, feet at shoulder width apart, dumbbells in both hands resting on front of the thigh.<br><br>**Exercise:**<br>While maintaining neutral spine position and slight knee bend, perform hip flexion by pushing the hips back and dragging the dumbbells along the front leg. Keep pushing the hips back until the dumbbells reach below the kneecaps, then extend the hips back by pushing them forward, maintaining the dumbbells' contact with the legs.

Box squat	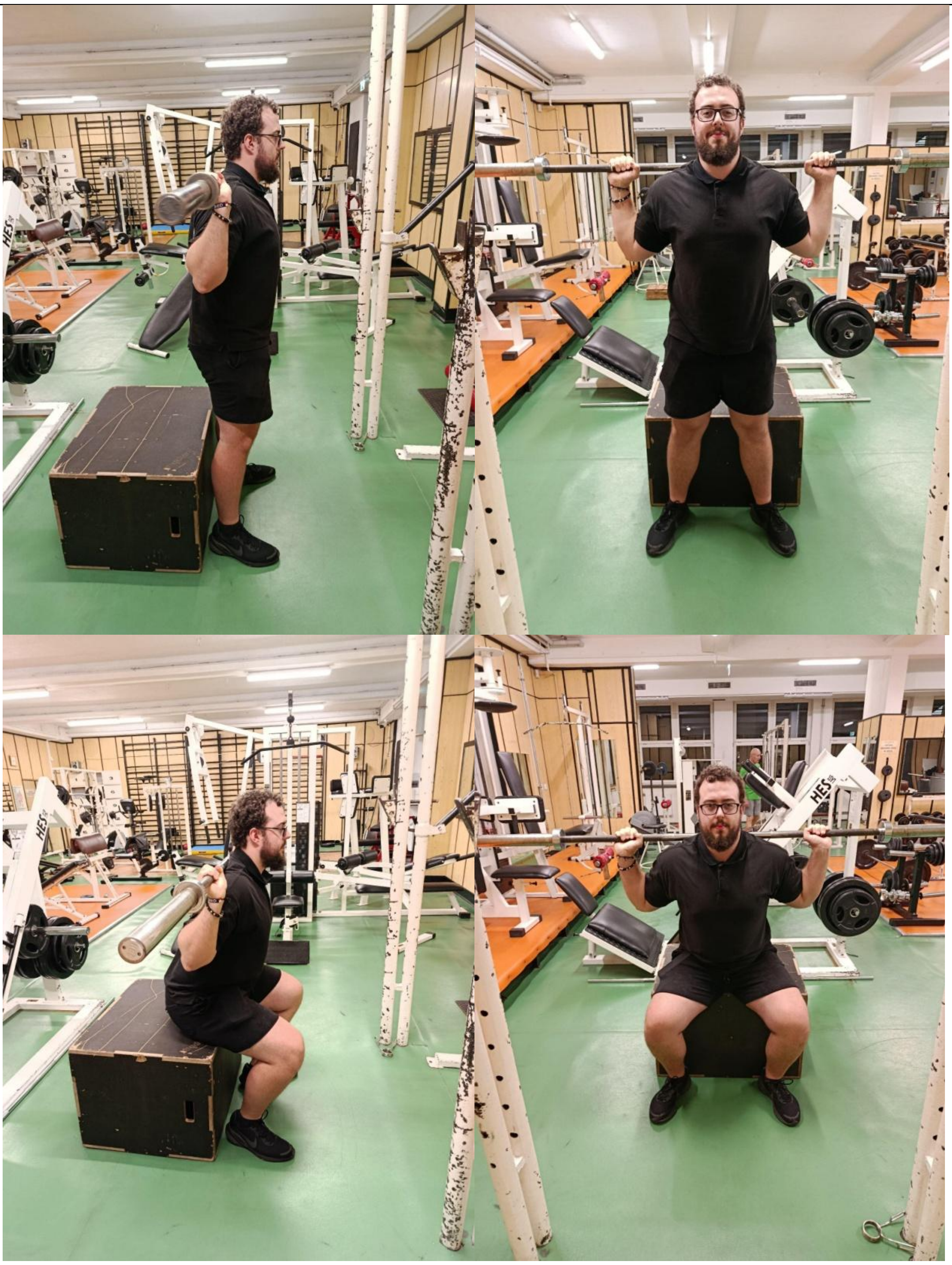<br>**Initial position:**<br>Set the box and the barbell racks to desirable height. Stand opposite to the barbell and grip it symmetrically to your liking. Duck your head underneath the middle of the bar and situate it on your upper trapezius muscles. Then, unrack the bar by extending your hips and knees. After stabilizing the position after unrack, take two to three steps back to have the box directly underneath you.<br><br>**Exercise:**<br>Inhale slightly. Descend toward the box until you make contact and sit down. Do not relax fully. Stand back up. Exhale upon ascent.<br><br>**Exercise modifications:**<br>If the 20kg barbell is too heavy to perform the desired number of repetitions and/or the necessary bar position on the back causes discomfort or pain, perform box goblet squats instead. Goblet squat requires the use of a dumbbell held in front of our body.

Modified french press	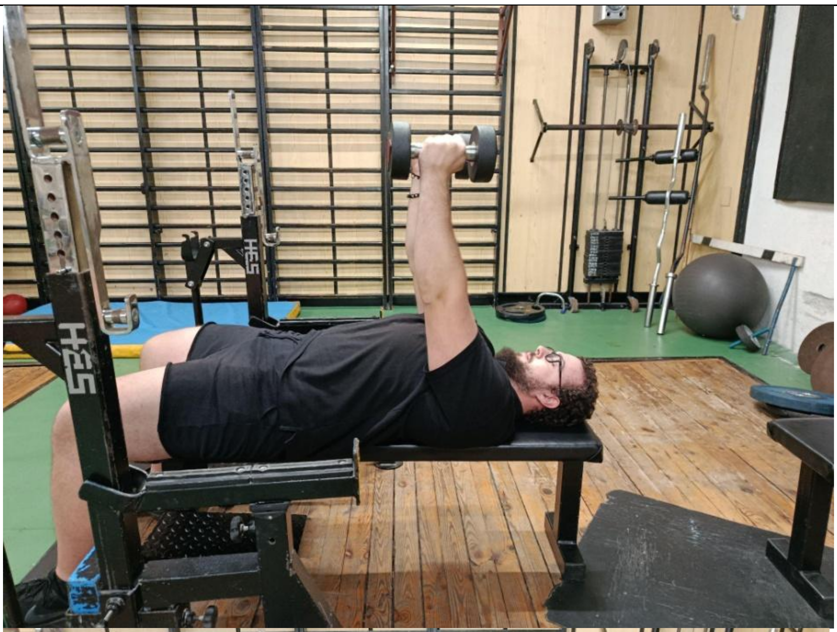<br>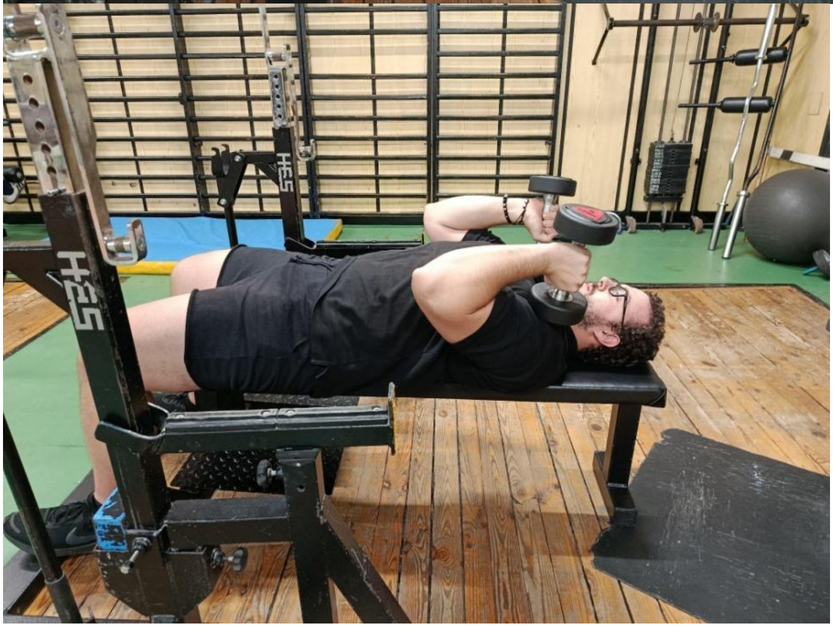<br>**Initial position:**<br>After grabbing two dumbbells, lay down flat facing upward on the bench. Situate your legs on the floor or on the bench. Do not overextend the lumbar spine. Extend your elbows and keep the dumbbells level with the shoulder joint at all times.<br><br>**Exercise:**<br>Perform simultaneous elbow flexion and shoulder extension by bringing the dumbbells to the shoulders. Upon making contact with the shoulders, perform elbow extension with shoulder flexion to extend the arms back up while keeping the dumbbells at shoulder level.

Seated shoulder-width cable row

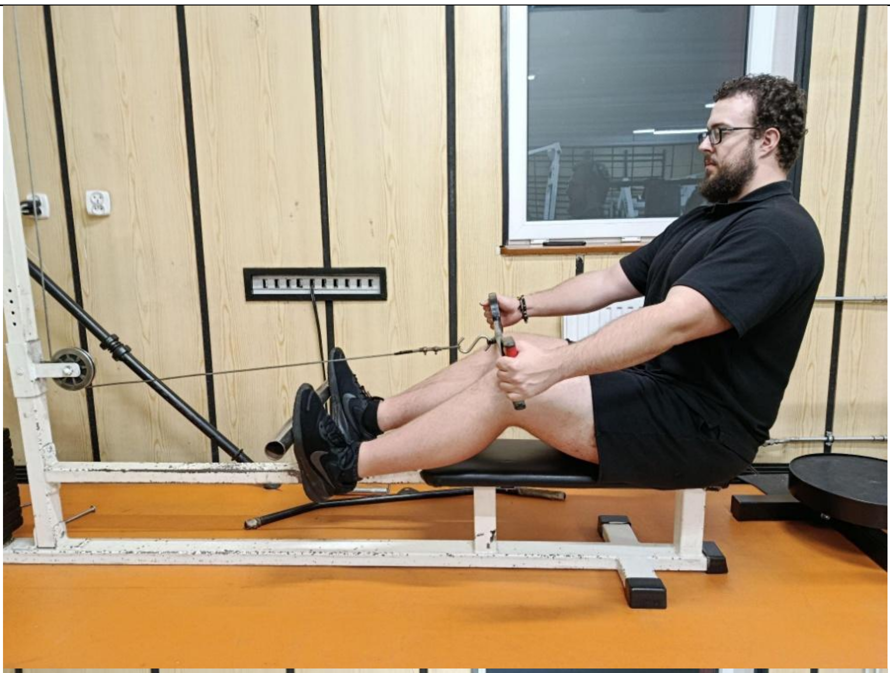

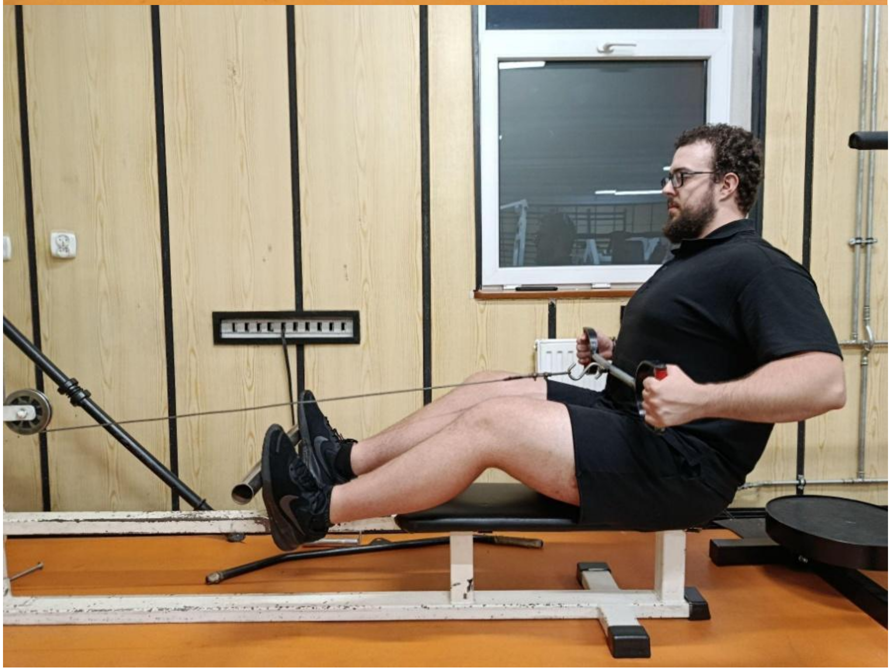

Initial position:

Grab onto the attachment symmetrically. Push your feet against the bar - try to achieve full knee extension to secure a stable position. Sit upright with spine in a neutral position.

Exercise:

Perform shoulder extension and elbow flexion by pulling the elbows back until the bar makes contact with the body. Aim for the bellybutton area. After the attachment makes contact with the body, perform elbow extension and shoulder flexion until arms are fully extended. Torso angle should remain unchanged throughout the set.

Exercise modifications:

Based on individual anthropometrical differences, we should assess the shoulder width of the subject in order to properly choose the rowing attachment. People with the wider frame should use the wider attachment, since narrow attachment will force the humerus to rotate inward at the end position, changing the outcome significantly. People with narrower frame should opt for the narrow attachment, since the wide attachment will force their elbows outward, changing the outcome significantly.

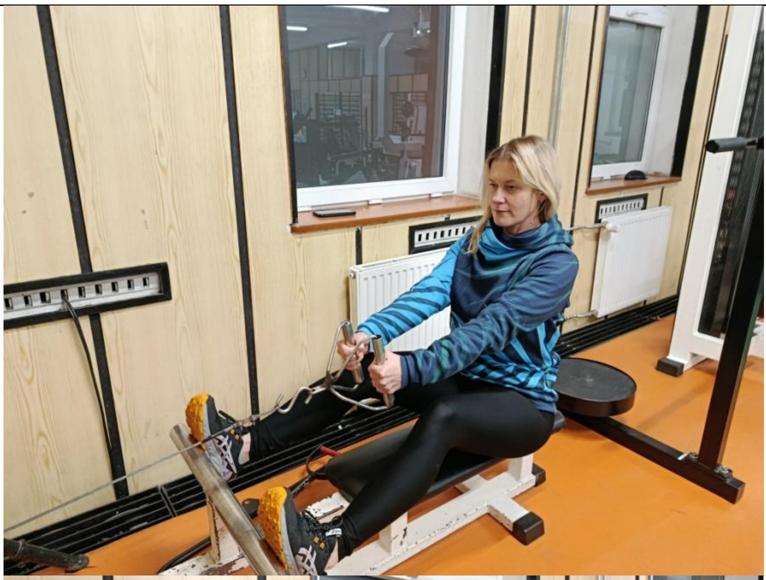

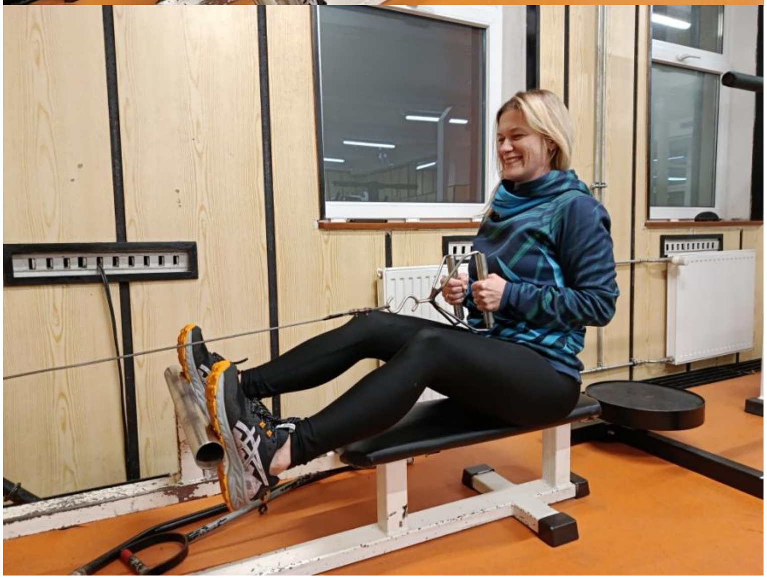

Exercise:

Perform shoulder extension and elbow flexion by pulling the elbows back until the bar makes contact with the body. Aim for the bellybutton area. After the attachment makes contact with the body, perform elbow extension and shoulder flexion until arms are fully extended. Torso angle should remain unchanged throughout the set.

Exercise modifications:

Based on individual anthropometrical differences, we should assess the shoulder width of the subject in order to properly choose the rowing attachment. People with the wider frame should use the wider attachment, since narrow attachment will force the humerus to rotate inward at the end position, changing the outcome significantly. People with narrower frame should opt for the narrow attachment, since the wide attachment will force their elbows outward, changing the outcome significantly.
