# Supplementary material for: Supervised 16-Week Multicomponent Exercise Training Programme for 18–55-Year-Old People Living with and Beyond Cancer—CONSORT 2025-Based Study Protocol of the Pilot Onco-Move Randomized Controlled Trial
Source: Nutrients. 2025 Dec 27;18(1):100. doi: 10.3390/nu18010100 (PMC12787456; doi:10.3390/nu18010100)
Supplement: Supplementary file 1 [file nutrients-18-00100-s001.zip › Table S3_Onco Move Equipment list.pdf]

Table S3: Exercise equipment for the Onco-Move project.

Equipment (Brand)	Number	Characteristics	Photo
Cycling Ergometer Nautilus	11	Nautilus U627, Nautilus Inc., Vancouver, WA, USA	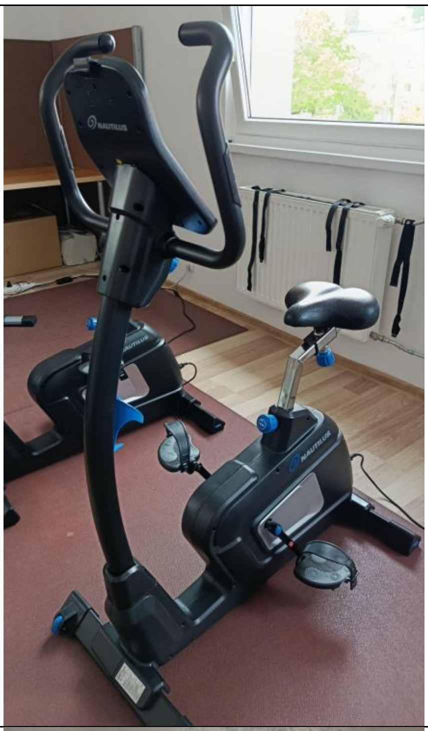
Sensory pillow MED+ (4Fizjo)	4	Colour: red<br>Measurement: 33 cm	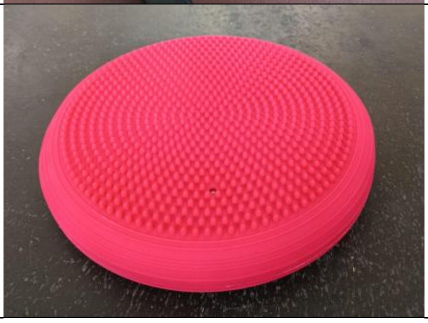
Cast Iron Dumbbells (4Fizjo)	8	Different colours<br>Weights: 1kg, 2kg, 3kg	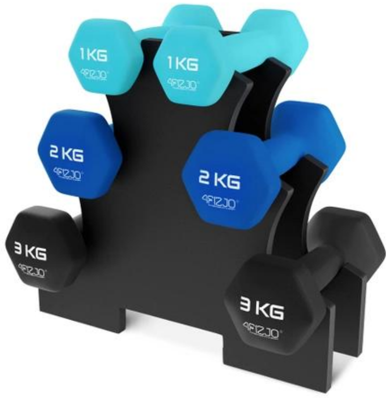

Rehabilitation Resistance Bands FLAT BAND (4Fizjo)	2	Different colours<br>Length: 2 m	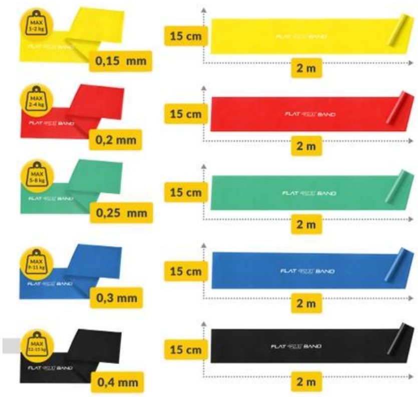
Exercise Bands MINI POWER BAND (4Fizjo)	8	Different colours<br>Measurements: 5 cm x 30 cm	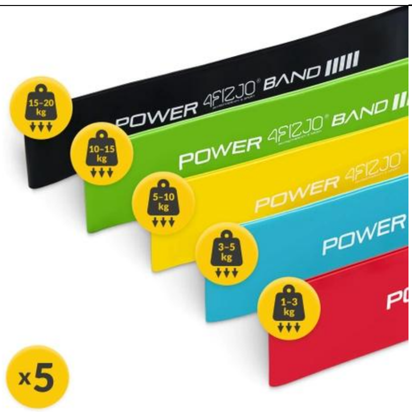
Pilates and Rehabilitation Ball (4Fizjo)	4	Colour: green<br>Size: 22 cm	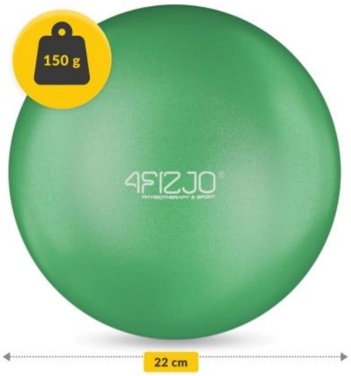
Dumbbell Set	1	Various Weights	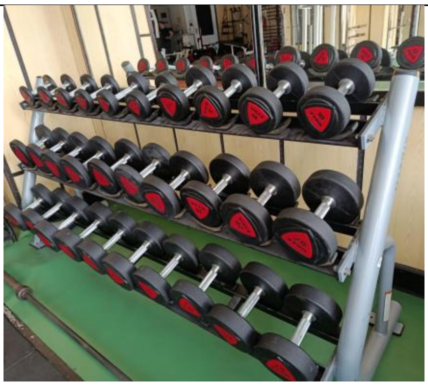

Plyometric box	1	Wooden<br>Measurements:<br>50 x 60 x 75 cm	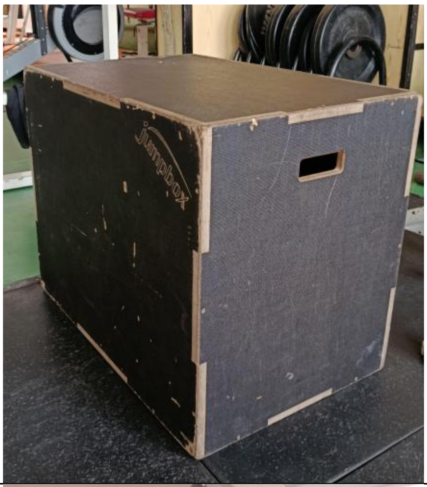
Dual-function machine by HES for training the latissimus dorsi muscles.	1	Featuring both a high and a low pulley system.	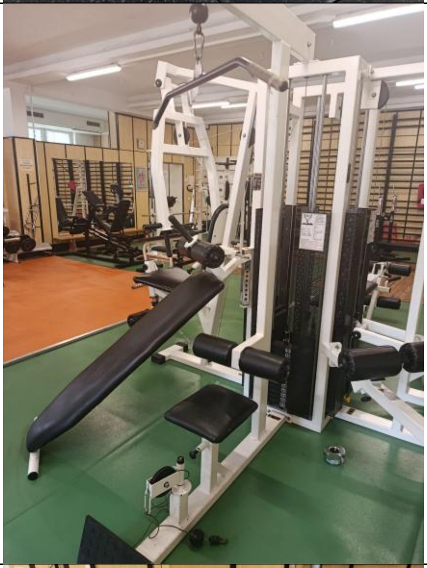
Hyperextension bench	1	90 degree angle, non-adjustable	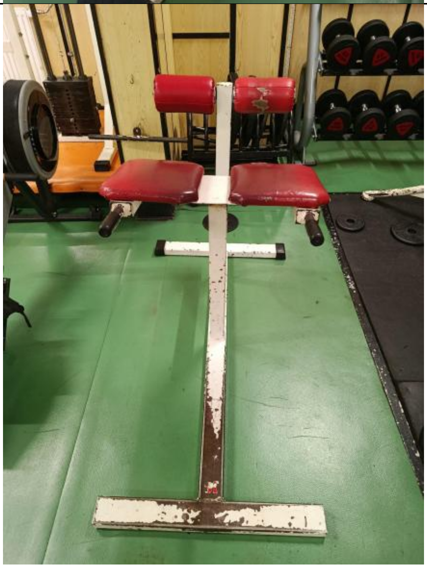

			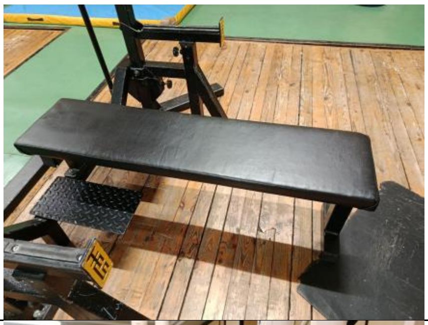
HES 20kg Olympic barbell	1	20kg<br>220cm length<br>28mm diameter	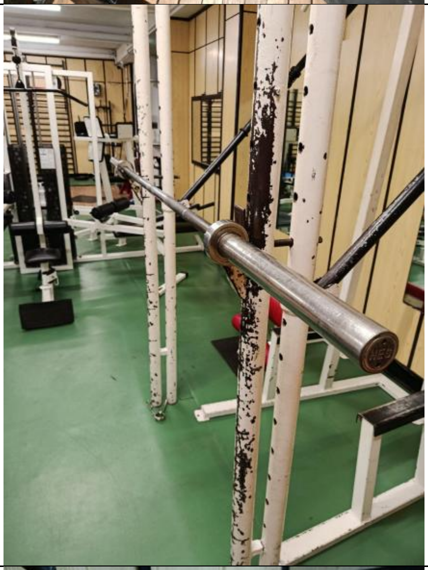
Narrower grip row attachment	1	Fixed width, rolling handles	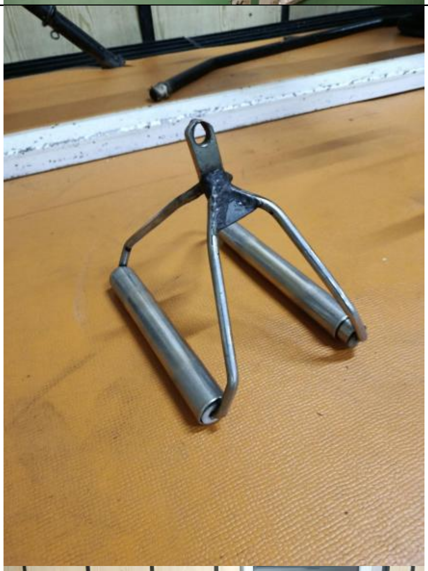
Seated horizontal pulley	1	Adjustable weight, non-adjustable height	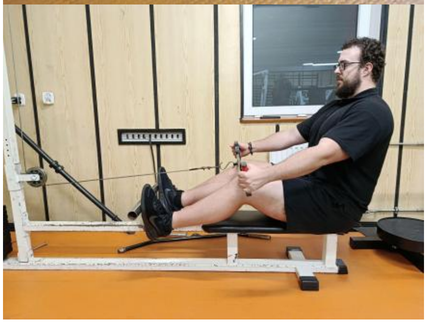

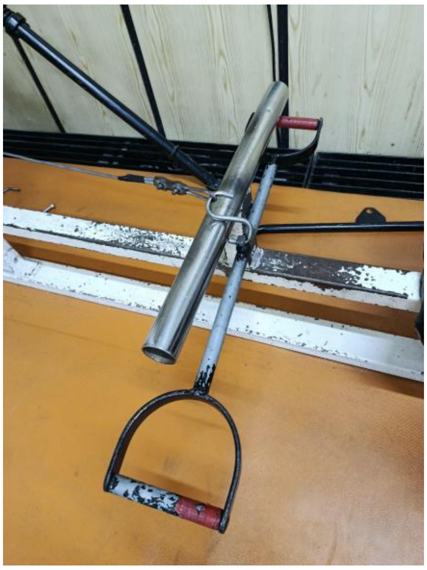

Wider grip row attachment

1 Fixed width, rolling handles
